# Pitfalls in Detecting *MET* Exon 14 Skipping Variants by DNA- and RNA-Based Next-Generation Sequencing Technologies in a Large Real-World Cohort and Results of the First Multinational External Quality Assessment Schemes

**DOI:** 10.1016/j.jmoldx.2026.05.007

**Published:** 2026-06-04

**Authors:** Carina Heydt, Michaela A. Ihle, Katharina Ilm, Jan Rehker, Peony Poon, Janna Siemanowski-Hrach, Roberto Pappesch, Svenja Wagener-Ryczek, Christoph Jonas, Jana Fassunke, Richard Riedel, Anne M. Schultheis, Reinhard Buettner, Sabine Merkelbach-Bruse, Udo Siebolts

**Affiliations:** ∗ Institute of Pathology, Faculty of Medicine and University Hospital Cologne, University of Cologne, Cologne, Germany; † Qualitätssicherungs-Initiative Pathologie (QuIP–Quality in Pathology) GmbH, Berlin, Germany; ‡ Institute of Pathology, School of Medicine and Health, Technical University Munich, Munich, Germany; § Center for Integrated Oncology, Department I of Internal Medicine, Faculty of Medicine and University Hospital Cologne, University of Cologne, Cologne, Germany; ¶ Lung Cancer Group Cologne, Cologne, Germany; ‖ Institute for Surgical Pathology, Medical Centre–University of Freiburg, Faculty of Medicine, University of Freiburg, Freiburg, Germany

## Abstract

*MET* exon 14 skipping mutations in non–small-cell lung cancer (NSCLC) are important biomarkers for targeted therapy, making accurate detection essential. This study analyzed 379 NSCLCs with mutations in *MET* exon 14 and adjacent splice sites using DNA- and RNA-based next-generation sequencing (NGS). The 379 samples contained 171 distinct mutations around *MET* exon 14, highlighting the diversity; 114 mutations were analyzed with a DNA- and an RNA-based NGS assay. Two large deletions and one synonymous splice variant causing exon 14 skipping were only detected by RNA-based NGS. Seven single-nucleotide variations in *MET* exon 14 did not induce skipping. A total of 57 variants could not be analyzed by RNA-based NGS because of insufficient material or RNA quality; among them, 18 were previously reported as skipping mutations, 30 were splice-site insertions/deletions, and 9 remained unclassified. Additionally, data from the first multinational external quality assessment schemes for *MET* exon 14 skipping mutation testing in formalin-fixed, paraffin-embedded tissue and liquid biopsies, organized by the lead panel institute and Quality in Pathology GmbH, are presented. High success rates (98%) for tissue are shown, whereas liquid biopsy tests had lower rates (37.5% in 2022, and 63% in 2024), primarily because of low allelic fractions and intronic deletions. This study highlights the importance of analyzing both DNA and RNA, urging improvements in sensitivity, coverage, and bioinformatics.

*MET* exon 14 skipping mutations occur in 3% to 4% of non–small-cell lung cancers (NSCLCs) and are heterogeneous.^1^ They have become an important actionable biomarker in patients with NSCLC. Tyrosine kinase inhibitors tepotinib and capmatinib received approval by the US Food and Drug Administration and European Medicines Agency for patients with advanced NSCLC with *MET* exon 14 skipping mutations.^2^^,^^3^

*MET* exon 14 skipping mutations first gained attention in 2015 when large studies reported patients with stage IV lung adenocarcinomas harboring a variety of *MET* exon 14 splice variants, which resulted in *MET* exon 14 loss and by that activation of MET proto-oncogene, receptor tyrosine kinase (MET).^4^^,^^5^

To date, >100 different mutations that can lead to *MET* exon 14 skipping have been described.^6^ They include single-nucleotide variations, deletions, insertions, combined deletions/insertions, and duplications that disrupt either the splice acceptor or the polypyrimidine tract at the 5′ end of *MET* exon 14 or the splice donor site at the 3′ end of *MET* exon 14.^7^

*MET* exon 14 skipping mutations can be detected on either the DNA or the RNA level. Currently, amplicon- or hybridization-based next-generation sequencing (NGS) assays are most commonly used for the detection of *MET* exon 14 skipping mutations. At the DNA level, the exact mutation leading to *MET* exon 14 skipping can be specified according to the Human Genome Variation Society (HGVS) nomenclature; however, the splicing effect, the loss of exon 14 transcription, and the generation of an intact fusion product can only be assessed by an RNA-based assay or by functional studies.^1^^,^^8^^,^^9^ For the reliable detection of all *MET* exon 14 skipping mutations, quality assured and sensitive detection methods are needed. Methodological standardization as well as internal and external quality assessment are utmost important to ensure accuracy and reproducibility of molecular testing, especially when decentralized testing is performed.^10^

In this study, a real-world cohort of 379 NSCLC samples from the years 2016 to 2024 with mutations in the splice sites of *MET* exon 14 as well as *MET* exon 14 were analyzed by DNA- and RNA-based NGS approaches, and the pitfalls of both methods were addressed. Additionally, data from the first multinational external quality assessment (EQA) schemes for molecular testing of *MET* exon 14 skipping mutations in formalin-fixed, paraffin-embedded (FFPE) tissue and liquid biopsy samples, conducted together with the Quality in Pathology (QuIP) GmbH (Berlin, Germany), will be presented.

## Materials and Methods

### Tumor Samples

A collection of 379 FFPE NSCLC tissue samples, previously tested positive for mutations in *MET* exon 14 and the splice sites of *MET* exon 14 during molecular pathologic routine diagnostics, were retrieved. The samples were selected from 2016 until 2024 from the registry of the Institute of Pathology of the University Hospital Cologne (Cologne, Germany). All samples were routinely prepared according to local practice. All methods were performed in accordance with the relevant guidelines and regulations of the Deutsche Akkreditierungsstelle.

The tumor type of all samples was determined as NSCLC by experienced pathologists (A.M.S., R.B., and U.S., among others). Tumor content of the samples was at least 10% or higher, and the necrotic portion was <10%.

FFPE tissue samples were obtained as part of routine clinical care under approved ethical protocols complied with the Ethics Committee of the Medical Faculty of the University of Cologne, and the same Ethics Committee waved the need for ethical approval (Ethics number 22-1265).

### Nucleic Acid Extraction

For DNA and total nucleic acid (tNA) extraction, sections (10 μm thick) were cut from FFPE tissue blocks. Sections were deparaffinized, and the tumor areas were macrodissected from unstained slides using a marked hematoxylin-eosin–stained slide as a reference.

For DNA extraction, samples were digested overnight using proteinase K, and DNA was isolated with the Maxwell 16 FFPE Plus Tissue LEV DNA Purification Kit (Promega, Mannheim, Germany) on the Maxwell 16 (Promega) or the Maxwell RSC FFPE Plus DNA Kit (Promega) on the Maxwell RSC (Promega) following manufacturer's instructions. For tNA extraction, the Maxwell RSC RNA FFPE Kit (Promega) was used on the Maxwell RSC (Promega) according to the manufacturer's instruction, and only the DNase solution during the digestion step was replaced by 50 μL water, as previously described.^11^

### Custom Amplicon-Based DNA NGS Assay

From 2016 until 2021, custom lung cancer–specific amplicon-based NGS assays (DNA-based amplicon NGS assay) were used for the detection of *MET* exon 14 mutations on the DNA level. *MET* intron 13 to intron 14 was covered (hg19: chromosome 7:116411694–116412154). The DNA content was measured using an in-house quantitative real-time PCR kit (GoTaq qPCR Master Mix; Promega), as previously described.^12^

For library preparation, either a custom GeneRead DNAseq Targeted Panel V2 and the GeneRead DNAseq Panel PCR Kit V2 (Qiagen, Hilden, Germany) or an Ion AmpliSeq Custom DNA Panel (Thermo Fisher Scientific, Waltham, MA) and the Ion AmpliSeq Library Kit 2.0 (Thermo Fisher Scientific) were used, as previously described.^12^ Libraries were sequenced on a MiSeq (Illumina, San Diego, CA) or NextSeq 500 (Illumina) following the manufacturer's recommendations. Data were exported as FASTQ files. Alignment and annotation were done using a modified version of a previously described method.^13^ A 5% cutoff for variant calls was used, and results were only interpreted if the coverage was >200×. For variant calling in *MET*, the transcript NM_001127500 (*https://www.ncbi.nlm.nih.gov/nuccore/NM_001127500*, last accessed May 6, 2026) was used.

### Custom Hybridization-Based DNA NGS Assay

From 2021 until now, a custom lung cancer–specific hybridization-based NGS assay (DNA-based hybridization NGS assay) was used. *MET* intron 13 to intron 14 was covered (hg38: chromosome 7:116771654–116772090). The DNA content was measured using a Tecan Infinite 200 microplate reader (Tecan, Maennedorf, Switzerland) and the QuantiFluor ONE dsDNA Kit (Promega).

For library preparation, a custom DNA-based hybridization NGS assay from Twist Bioscience (South San Francisco, CA) was used, according to the manufacturer's instruction. The Twist Library Preparation EF Kit (Twist Bioscience) and Twist UMI Adapter System (Twist Bioscience) were used for enzymatic fragmentation, end repair, dA tailing, adapter ligation, and library amplification. In both library amplification steps, the Twist enzyme was replaced by the KAPA HIFI HotStart Ready Mix (Roche, Basel, Switzerland). For target enrichment and hybridization, custom biotinylated probes (Twist Bioscience), the Twist Universal Blockers (Twist Bioscience), the Twist Binding and Purification Beads (Twist Bioscience), as well as the Twist Hybridization and Wash Kit (Twist Bioscience) were applied. Postenriched libraries were quantified, pooled, and sequenced on a NextSeq 500 or NovaSeq (Illumina). Data were exported as FASTQ files. Alignment and annotation were done using an in-house pipeline. Sequencing data were stripped from adapters by fastp,^14^ followed by primary alignment via bwa mem version 0.7.17^15^ and sorting with sambamba.^16^ Read grouping and calling of molecular consensus reads were accomplished with fg-bio tools version 2.2.2 (*https://fulcrumgenomics.github.io/fgbio*). Read groups were converted back to FASTQ with bedtools bamtofastq^17^ and realigned with bwa mem version 0.7.17. Variants were called with Genome Analysis Toolkit version 4.1.9.0 Mutect2.^18^^,^^19^ Raw calls were annotated with ANNOVAR.^20^^,^^21^ ANNOVAR was run with a splicing threshold of 1500 bp from *MET* exon 14 exon/intron borders. Annotated variant call format (vcf) files were filtered with bcftools^22^ based on a depth >10 reads a variant allele frequency >0.001 and an annotation that specified the variant as either exonic or splicing. Another instance of bcftools removed variants that were not annotated as splicing but coding synonymous. For intronic *MET* exon 14 skipping variants, all calls that surpassed a depth of 10 reads were considered. The CollectHsMetrics tool was used to determine coverage.^23^ A 5% cutoff for variant calls was used, and results were only interpreted if the coverage was >200×. For variant calling in *MET*, the transcript NM_001127500 (*https://www.ncbi.nlm.nih.gov/nuccore/NM_001127500*, last accessed May 6, 2026) was used.

### RNA-Based NGS Assay

Total nucleic acid extracts were quantified with the Qubit RNA BR Assay Kit (Thermo Fisher Scientific) on the Qubit 2.0 Fluorometer (Thermo Fisher Scientific).

For the analysis of *MET* exon 14 skipping mutations on the RNA level, the Archer FusionPlex CTL assay (2017 to 2018) and the Archer FusionPlex Lung assay (2018 to 2022) as well as the Archer FusionPlex Lung version 2 assay (from 2023; IDT, Coralville, IA) were used, according to the manufacturer's instructions. The Archer assay was performed as a reflex follow-up analysis in cases with an *MET* exon 14 skipping mutation detected at the DNA level or in the absence of identifiable driver alterations in *EGFR*, *KRAS*, *BRAF*, and *ERBB2* (*HER2*). Before the year 2017, only the amplicon-based DNA NGS assay was used. For this assay, up to 200 ng tNA input per sample was used for the preparation of each library. Purified libraries were quantified using the KAPA Library Quantification Kit (Roche) and pooled to equimolar concentrations. Sequencing was performed on the MiSeq System or NextSeq 500 System (Illumina), and results were analyzed using the Archer Analysis software version 5.1.3 until November 2019, Archer Analysis software version 6.2.1 until June 2021, followed by Archer Analysis software version 6.2.7 (ArcherDX; IDT) with the following quality parameters: on-target rate >85%, RNA all fragments >200,000, average unique RNA start sites per gene-specific primer 2 (GSP2) control >10, RNA fragment lengths >100, and RNA fragment housekeeping genes >100.

### Sensitivity Analysis of the RNA-Based NGS Assay

Sensitivity of the Archer FusionPlex Lung assay (IDT) for the evaluation of *MET* exon 14 skipping mutations was determined by a serial dilution of the Seraseq FFPE Tumor Fusion RNA version 4 Reference Material (*MET* exon 14 skipping; HiSS Diagnostics GmbH, Freiburg, Germany) and the Seraseq FFPE WT (DNA/RNA) Reference Material (wild type; HiSS Diagnostics GmbH). The 200-, 50-, 25-, 10-, 5-, 1-, and 0-ng mutated standard were mixed with wild-type standard to achieve a final amount of 200 ng tNA. Dilutions were sequenced with the Archer FusionPlex Lung assay (IDT), as described in RNA-Based NGS Assay.

### EQA Schemes for Molecular Testing of *MET* Exon 14 Skipping Organized by QuIP GmbH

The first multinational EQA schemes for the assessment of *MET* exon 14 skipping mutations in FFPE tissue and liquid biopsy were designed together with the QuIP GmbH. The EQA schemes were structured into internal validation phase and external testing phase for participating pathology institutes. Reference samples for the EQA sub-scheme for FFPE tissue were chosen and tested by the lead panel institute (Institute of Pathology, University Hospital Cologne) based on the mutational spectrum of *MET* exon 14 skipping mutations and their prevalence. In the internal validation phase, six panel institutes performed cross-validation testing of the chosen material (10 cases), whereby different methods for the detection on DNA and RNA level were used. On the basis of the results of the cross-validation testing, the suitable cases were selected and target values were defined. The EQA sub-schemes for liquid biopsy samples were performed with 10 different artificial samples (SensID GmbH, Rostock, Germany). Each sample contained 60 ng of either wild-type DNA or a combination of wild-type and mutated fragmented DNA in different allelic fractions spiked in 2 mL of human nucleic acid–free plasma. The composition was defined by the lead panel institute and based on the mutational spectrum of *MET* exon 14 skipping mutations. In the internal validation phase, each sample was cross-validated by the lead panel institute and two additional panel institutes by using different NGS panels.

After successful completion of the internal validation phase, the external open EQA sub-scheme was advertised on the QuIP GmbH homepage, and pathology institutes could register. Institutes received the corresponding material and had 10 to 15 working days for submitting their tests results.

### EQA Sub-Scheme with FFPE Tissue

For the external testing phase of the EQA sub-scheme with FFPE tissue, a total of 50 institutes from Germany, Austria, and Switzerland registered and submitted results.

The FFPE tissue was obtained from archived resected tumor blocks from NSCLC primary tumors or resected metastases. From 10 suitable and pretested cases (internal validation phase) (Table 1), three slides per case with tissue material were sent to the participants, two 10-μm–thick sections for nucleic acid extraction and one 2- to 3-μm–thick section for hematoxylin and eosin staining.

**Table 1:** Reference Values of Cases 1 to 10 for *MET* Exon 14 Skipping EQA Sub-Scheme with FFPE Material

Case no.	Split 1		Split 2		Split 3	
	*MET* exon14 skipping	Variant (AF, %)	*MET* exon14 skipping	Variant (AF, %)	*MET* exon14 skipping	Variant (AF, %)
1	No	WT	Yes	c.3082+2T>C (28.5)	Yes	c.3082G>A p.Asp1028Asn (15.4)
2	Yes	c.3082+3A>G (36)	No	WT	No	WT
3	No	WT	No	WT	Yes	c.3082+3_ 3082+32del (27)
4	No	WT	Yes	c.2942-26_ 2942-10del (12.9)	No	WT
5	No	WT	No	WT	Yes	c.2942–20_ 2942-9del (56.1)
6	No	WT	Yes	c.2942-18_ 2942-6del (16)	No	WT
7	Yes	c.3082+1G>A (22.3)	No	WT	No	WT
8	No	WT	No	WT	Yes	c.3082+1G>A (19.7)
9	Yes	c.2942-13_ 2945del (15.8)	No	WT	No	WT
10	Yes	c.2942-26_ 2942-10del (12.9)	Yes	c.3082+1G>A (9.6)	No	WT

Data are from NM_001127500 (*https://www.ncbi.nlm.nih.gov/nuccore/NM_001127500*, last accessed May 6, 2026). Participants received split 1, 2, or 3.

AF, allelic fraction; c, cDNA level; EQA, external quality assessment; FFPE, formalin fixed, paraffin embedded; p, protein level; WT, wild type.

The material was selected on the basis of the following criteria:

• Resected NSCLC specimens to have sufficient and consistent material available for all participants
• Mutations with an allelic fraction of at least 5%
• Both single-nucleotide variations and deletions, insertions, or combined deletions/insertions that lead to *MET* exon 14 skipping

The mutations were named according to the reference sequence NM_001127500 (*https://www.ncbi.nlm.nih.gov/nuccore/NM_001127500*, last accessed May 6, 2026) and in concordance with the HGVS (*https://hgvs-nomenclature.org/stable*, last accessed May 6, 2026). In the multinational EQA sub-scheme, the institutes were free to choose whether to perform the detection of *MET* exon 14 skipping using DNA- and/or RNA-based methods. Unfortunately, because of limited material, twice the amount of tissue sections could not be provided for participants who wanted to perform both DNA- and/or RNA-based methods, and thus the optimal amount of tumor material may not have been available for the extraction of DNA and RNA. The EQA sub-scheme had to be divided into three section splits because of limited material, and not all participants received the same samples. The mutational spectrum was comparable in the three splits for all participants (Table 1). Each case was cross-validated by the lead panel institute and two panel institutes. The minimal allelic fraction and the region to be analyzed for *MET* exon 14 skipping were stated in the instructions of the EQA sub-scheme so that each participant could check whether the established test method would be suitable. The participants had 10 working days to perform the analyses and report the results back to the QuIP GmbH online.

The participants had to determine the *MET* mutation status in intron 13, exon 14, and intron 14 of *MET*, which could cause *MET* exon 14 skipping using molecular methods. Two points were awarded for the correct determination of the *MET* mutation status per case (mutation detected: yes or no). For the 10 samples, a maximum of 20 points could be achieved, and a minimum of 19 points was required to pass the EQA sub-scheme. One point was awarded if a sample could not be analyzed because of technical reasons. However, this option could only be used once. Information on the exact mutation on coding and protein level according to the HGVS and the allelic fraction was not mandatory but could be given optional.

### EQA Sub-Scheme for Liquid Biopsy Samples

Two liquid biopsy EQA sub-schemes were conducted, one in 2022 and one in 2024.

In 2022, 13 test sets were requested but only eight participants submitted their results. In 2024, 18 participants registered, and 16 participants submitted results.

The liquid biopsy samples were produced and shipped to the participants by SensID GmbH. The clinically relevant mutation sequences were synthesized as high-fidelity double-stranded DNA fragments with a size distribution similar to cell-free DNA/circulating tumor DNA by an European Norm (EN) International Organization for Standardization (ISO) 13485–certified service provider. The participants received 10 different samples. Each sample contained 60 ng of either wild-type DNA or a combination of wild-type and mutated fragmented DNA in different allele frequencies spiked in 2 mL of human nucleic acid–free plasma.

The reference values of the test material for each case are shown in Tables 2 and 3. The allelic fractions were determined by SensID GmbH using droplet digital PCR (Tables 2 and 3).

**Table 2:** Reference Values of Cases 1 to 10 for *MET* Exon 14 Skipping EQA Sub-Scheme with Liquid Biopsy Samples in 2022

Case no.	*MET* exon 14 skipping detectable?	Designation according to HGVS	Allelic fraction, % (according to manufacturer)
1	Yes	c.3082+1G>T	4.96
2	Yes	Wild type	0.0
3	Yes	c.3082G>C; (p.Asp1028His)	4.85
4	No	c.2942-26_2942-10del	2.03
5	Yes	c.2942-18_2942-5del	2.06
6	Yes	c.3082+1G>T	2.34
7	No	c.2942-26_2942-10del	9.82
8	Yes	Wild type	0.0
9	Yes	c.2942-18_2942-5del	10.95
10	Yes	c.3082G>C; (p.Asp1028His)	2.16

c, cDNA level; EQA, external quality assessment; HGVS, Human Genome Variation Society; p, protein level.

**Table 3:** Reference Values of Cases 1 to 10 for *MET* Exon 14 Skipping EQA Sub-Scheme with Liquid Biopsy Samples in 2024

Case no.	*MET* exon 14 skipping detectable?	Designation according to HGVS	Allelic fraction, % (according to manufacturer)
1	Yes	c.2942-26_2942-10del	2.03
2	Yes	c.3082G>C; (p.Asp1028His)	2.16
3	Yes	c.2942-18_2942-5del	10.95
4	No	Wild type	0.0
5	Yes	c.3082+1G>T	2.34
6	Yes	c.2942-18_2942-5del	2.06
7	No	Wild type	0.0
8	Yes	c.3082G>C; (p.Asp1028His)	4.85
9	Yes	c.2942-26_2942-10del	9.82
10	Yes	c.3082+1G>T	4.96

c, cDNA level; EQA, external quality assessment; HGVS, Human Genome Variation Society; p, protein level.

The participants had to determine the *MET* mutation status in intron 13, exon 14, and intron 14 of *MET*, which could cause *MET* exon 14 skipping using molecular methods.

Two points were awarded for the correct determination of the *MET* mutation per case (mutation detected: yes or no). For the 10 samples, a maximum of 20 points could be achieved, and a minimum of 18 points was required to pass the EQA sub-scheme. The passing limit was lower compared with FFPE because of the samples with lower allelic fraction to be detected in the liquid biopsy. One point was awarded if a sample could not be analyzed for technical reasons. However, this option could only be used once.

In addition, the participants were asked to specify the detected mutation on c.DNA level according to the HGVS, but the annotation was excluded from assessment of successful participation.

## Results

### Sensitivity Analysis of the RNA-Based NGS Assay

**Figure 1:**
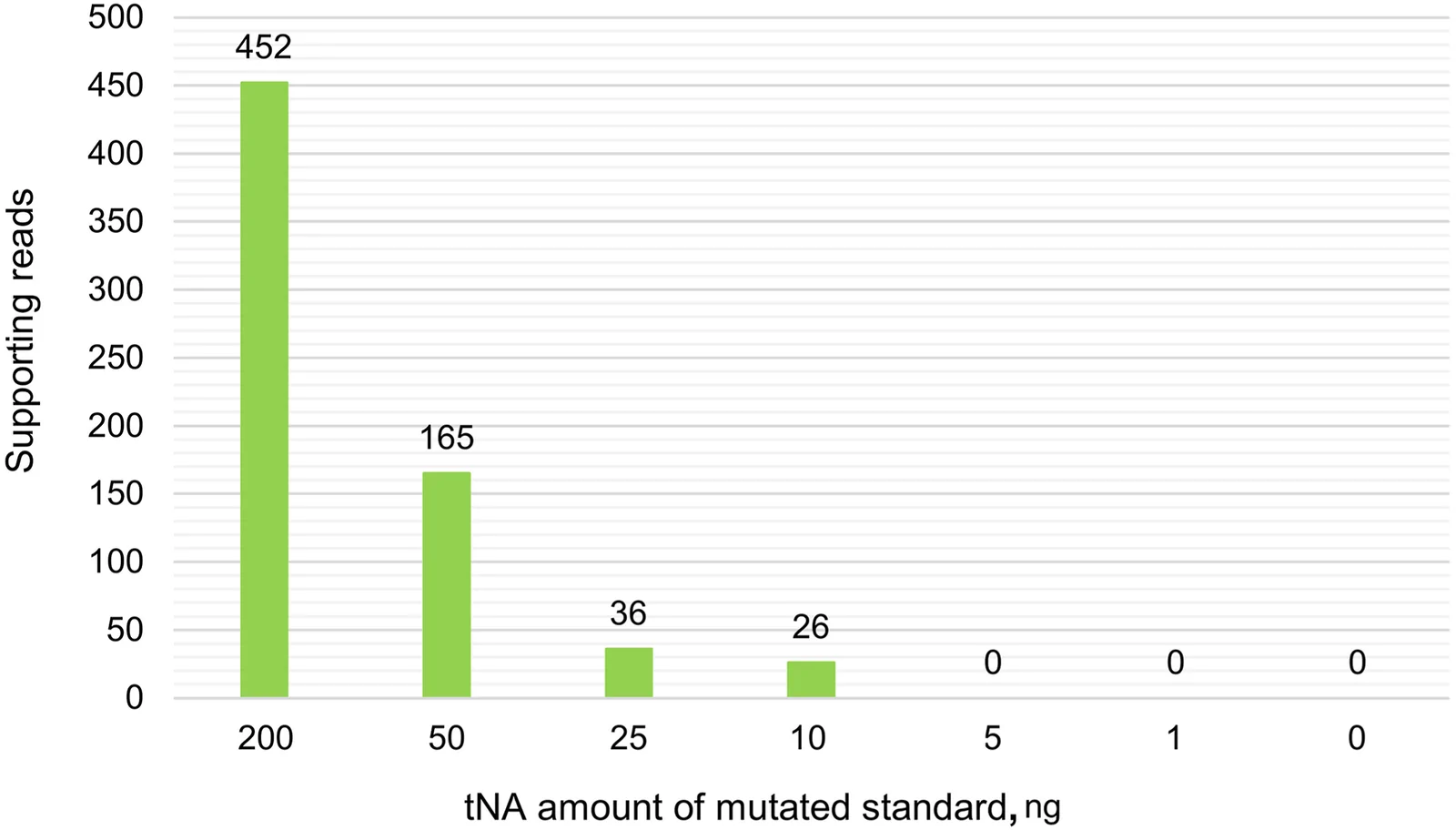
Sensitivity of the RNA-based next-generation sequencing assay determined by a serial dilution. Sensitivity of the Archer FusionPlex Lung assay (IDT) for the evaluation of *MET* exon 14 skipping mutation was determined by a serial dilution of the Seraseq FFPE Tumor Fusion RNA version 4 Reference Material (*MET* exon 14 skipping mutation; HiSS Diagnostics GmbH) with the Seraseq FFPE WT (DNA/RNA) Reference Material (wild type; HiSS Diagnostics GmbH). The 200-, 50-, 25-, 10-, 5-, 1-, and 0-ng mutated standards were mixed with wild-type standard to achieve a final amount of 200 ng total nucleic acid (tNA). The number of supporting reads was documented for each dilution.

The technical sensitivity of the Archer FusionPlex Lung assay (IDT) was determined by a serial dilution (Figure 1). This assay detected *MET* exon 14 skipping mutations up to a dilution of 1:20 of the commercial reference material. The recommended input amount of the Archer FusionPlex Lung assay (IDT) is 200 ng tNA regardless of the tumor cell content. The Archer FusionPlex Lung assay (IDT) had a limit of detection of 10 ng mutated tNA, representing 26 fusion-supporting reads in the experiment. This represented an allelic fraction of 5% and a tumor cell content of 10% if only one allele was mutated. Below 10 ng tumor tNA input, the Archer FusionPlex Lung assay (IDT) did not detect any fusion-supporting reads for *MET*(exon 13)::*MET*(exon 15).

### Real-World Cohort of 379 NSCLC Samples from the Years 2016 to 2024

**Figure 2:**
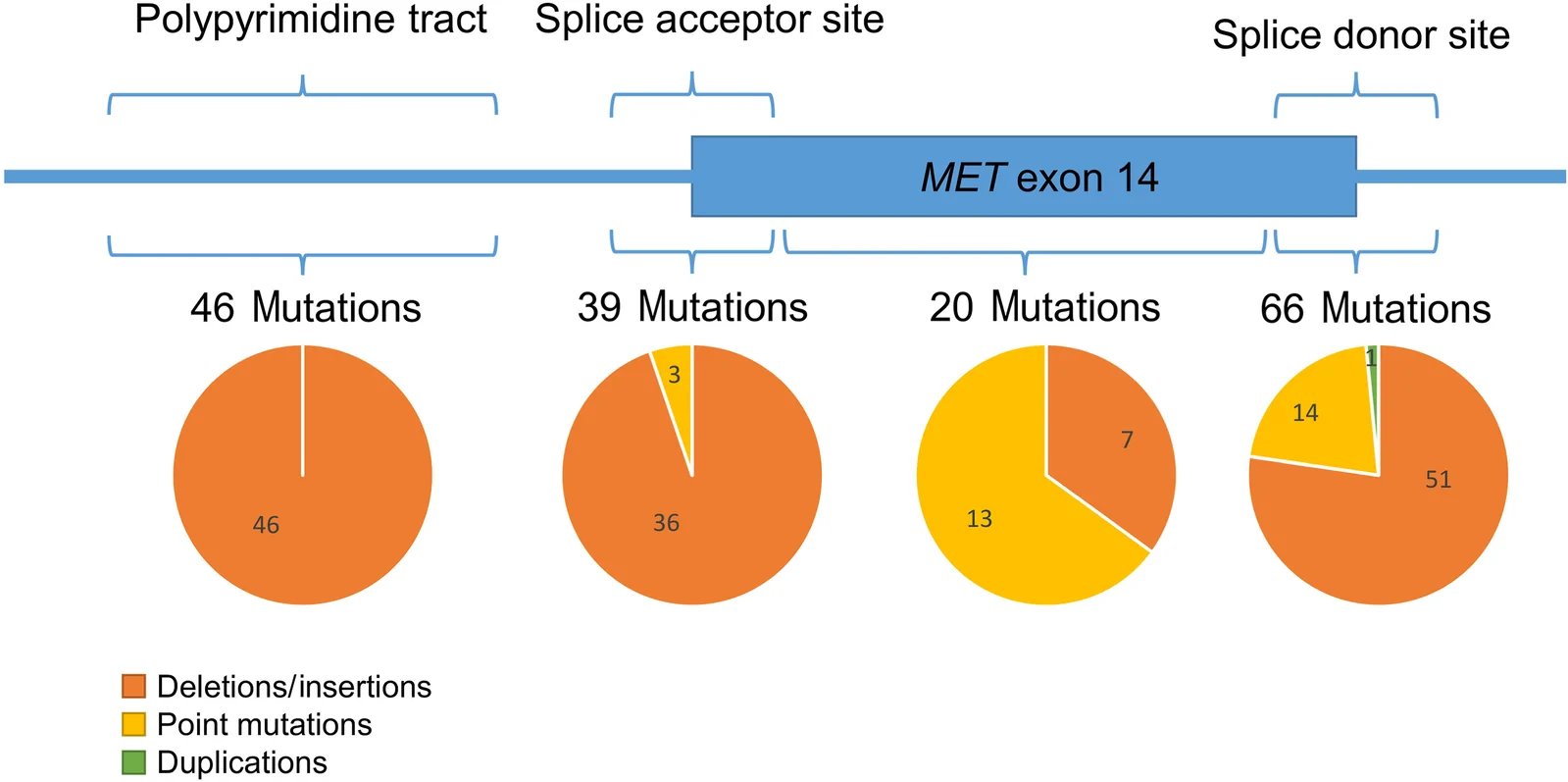
Classification and distribution of 171 different *MET* exon 14 mutations. Non–small-cell lung cancer samples detected in Cologne, Germany: mutations were deletions, insertions, point mutations, or duplications and located in the polypyrimidine tract, the spice acceptor site, in exon 14, or in the splice donor site.

A total of 379 NSCLC samples from individual patients with mutations in *MET* exon 14 and the splice sites of *MET* exon 14 were retrieved from the registry of the Institute of Pathology of the University Hospital Cologne. All samples were previously analyzed with a DNA- or an RNA-based NGS assay or a combination of both, spanning the region of *MET* exon 14 from intron 13 to intron 14 (hg38: chr7:116771654–116772090). *MET* exon 14 alterations were point mutations, deletions, insertions, combined deletions/insertions, or small duplications. The 379 NSCLCs had 171 different mutations in *MET* exon 14 and/or adjacent splice sites (Figure 2, Tables 4 and 5, and Supplemental Table S1^24^, ^25^, ^26^, ^27^). A total of 46 of the mutations were at the polypyrimidine tract, 39 of the mutations were at the splice acceptor site, 20 mutations were in exon 14, and 66 were at the splice donor site, showing the wide mutational spectrum of *MET* exon 14 skipping mutations (Figure 2).

**Table 4:** The 107 Mutations in the Splice Site of *MET* Exon 14 Leading to Exon 14 Skipping

Mutation detected by DNA-based NGS assay	No.	RNA-based NGS assay
*MET*: c.2942-60_2961del	1	*MET*(Ex.13)::*MET*(Ex.15)
***MET*****: c.2942-49_2963delinsC**	1	*MET*(Ex.13)::*MET*(Ex.15)
***MET*****: c.2942-47_2944del**	1	*MET*(Ex.13)::*MET*(Ex.15)
*MET*: c.2942-46_2942-30del	1	*MET*(Ex.13)::*MET*(Ex.15)
*MET*: c.2942-46_2942-18delinsT	1	*MET*(Ex.13)::*MET*(Ex.15)
*MET*: c.2942-43_2944del	1	*MET*(Ex.13)::*MET*(Ex.15)
*MET*: c.2942-38_2942-1del	1	*MET*(Ex.13)::*MET*(Ex.15)
*MET*: c.2942-37_2942–21del	1	*MET*(Ex.13)::*MET*(Ex.15)
*MET*: c.2942-36_2944del	1	*MET*(Ex.13)::*MET*(Ex.15)
*MET*: c.2942-32_2945del	1	*MET*(Ex.13)::*MET*(Ex.15)
*MET*: c.2942-32_2942-12del; *MET*: c.3005dup p.Ser1003Lysfs∗15	1	*MET*(Ex.13)::*MET*(Ex.15)
*MET*: c.2942-30_2942-6del	1	*MET*(Ex.13)::*MET*(Ex.15)
*MET*: c.2942-30_2942-3del	1	*MET*(Ex.13)::*MET*(Ex.15)
*MET*: c.2942-30_2942-25de	1	*MET*(Ex.13)::*MET*(Ex.15)
*MET*: c.2942-30_2942-16delinsC	1	*MET*(Ex.13)::*MET*(Ex.15)
*MET*: c.2942-28_2959del	1	*MET*(Ex.13)::*MET*(Ex.15)
*MET*: c.2942-28_2942-2del	2	*MET*(Ex.13)::*MET*(Ex.15)
*MET*: c.2942-28_2942-16del	1	*MET*(Ex.13)::*MET*(Ex.15)
*MET*: c.2942-27_2942-5del	1	*MET*(Ex.13)::*MET*(Ex.15)
*MET*: c.2942-27_2942-11del	1	*MET*(Ex.13)::*MET*(Ex.15)
*MET*: c.2942-26_2942-10del	1	*MET*(Ex.13)::*MET*(Ex.15)
*MET*: c.2942-24_2942-3del	2	*MET*(Ex.13)::*MET*(Ex.15)
*MET*: c.2942-23_2942-1del	1	*MET*(Ex.13)::*MET*(Ex.15)
*MET*: c.2942-22_2965del	1	*MET*(Ex.13)::*MET*(Ex.15)
*MET*: c.2942-21_2949del	1	*MET*(Ex.13)::*MET*(Ex.15)
*MET*: c.2942-21_2942-20delinsAAGA	1	*MET*(Ex.13)::*MET*(Ex.15)
*MET*: c.2942-20_2945del	4	*MET*(Ex.13)::*MET*(Ex.15)
*MET*: c.2942-20_2942-9del	1	*MET*(Ex.13)::*MET*(Ex.15)
*MET*: c.2942-20_2942-8delinsCTT	1	*MET*(Ex.13)::*MET*(Ex.15)
*MET*: c.2942-20_2942-5delinsC	1	*MET*(Ex.13)::*MET*(Ex.15)
*MET*: c.2942-20_2942-3del	1	*MET*(Ex.13)::*MET*(Ex.15)
*MET*: c.2942-20_2942-2del	2	*MET*(Ex.13)::*MET*(Ex.15)
*MET*: c.2942-20_2942–11del	3	*MET*(Ex.13)::*MET*(Ex.15)
*MET*: c.2942-19_2949delinsCCTTT; *MET*: c.3082G>C p.Asp1028His	1	*MET*(Ex.13)::*MET*(Ex.15)
*MET*: c.2942-19_2942-9del	1	*MET*(Ex.13)::*MET*(Ex.15)
*MET*: c.2942-18_2942-9del	1	*MET*(Ex.13)::*MET*(Ex.15)
*MET*: c.2942-18_2942-9del	1	*MET*(Ex.13)::*MET*(Ex.15)
*MET*: c.2942-18_2942-7del	1	*MET*(Ex.13)::*MET*(Ex.15)
*MET*: c.2942-18_2942-6del	1	*MET*(Ex.13)::*MET*(Ex.15)
*MET*: c.2942-18_2942-5del	2	*MET*(Ex.13)::*MET*(Ex.15)
*MET*: c.2942-18_2942-4del	2	*MET*(Ex.13)::*MET*(Ex.15)
*MET*: c.2942-18_2942-1del	1	*MET*(Ex.13)::*MET*(Ex.15)
*MET*: c.2942-18_2942-17insAGA	1	*MET*(Ex.13)::*MET*(Ex.15)
*MET*: c. [2942-18_2942-6del];[2942-16_2942-4del]	1	*MET*(Ex.13)::*MET*(Ex.15)
*MET*: c.2942-17_2942-9del	1	*MET*(Ex.13)::*MET*(Ex.15)
*MET*: c.2942-17_2942-4delinsA	1	*MET*(Ex.13)::*MET*(Ex.15)
*MET*: c.2942-17_2942-4del	1	*MET*(Ex.13)::*MET*(Ex.15)
*MET*: c.2942-16_2976delinsG	1	*MET*(Ex.13)::*MET*(Ex.15)
*MET*: c.2942-16_2942-1del	1	*MET*(Ex.13)::*MET*(Ex.15)
*MET*: c.2942-15_2945del	1	*MET*(Ex.13)::*MET*(Ex.15)
*MET*: c.2942-15_2942-4del	3	*MET*(Ex.13)::*MET*(Ex.15)
*MET*: c.2942-14_2942-4del	2	*MET*(Ex.13)::*MET*(Ex.15)
*MET*: c.2942-12_2965del	1	*MET*(Ex.13)::*MET*(Ex.15)
*MET*: c.2942-10C>A	1	*MET*(Ex.13)::*MET*(Ex.15)
*MET*: c.2942-9_2967del	1	*MET*(Ex.13)::*MET*(Ex.15)
*MET*: c.2942-5_2961del	3	*MET*(Ex.13)::*MET*(Ex.15)
*MET*: c.2942-4_2962delinsG	1	*MET*(Ex.13)::*MET*(Ex.15)
*MET*: c.2942-4_2961del	1	*MET*(Ex.13)::*MET*(Ex.15)
*MET*: c.2942-2_2960del	1	*MET*(Ex.13)::*MET*(Ex.15)
*MET*: c.2942-1G>C	2	*MET*(Ex.13)::*MET*(Ex.15)
*MET*: c.3059_3082+1del	1	*MET*(Ex.13)::*MET*(Ex.15)
*MET*: c.3062_3082+22del	1	*MET*(Ex.13)::*MET*(Ex.15)
*MET*: c.3063_3074delinsG p.Tyr1021Ter	1	*MET*(Ex.13)::*MET*(Ex.15)
*MET*: c.3063_3077del p.Tyr1021Ter	1	*MET*(Ex.13)::*MET*(Ex.15)
*MET*: c.3066_3081del p.Ala1023IlefsTer19	1	*MET*(Ex.13)::*MET*(Ex.15)
*MET*: c.3066_3082+1del	1	*MET*(Ex.13)::*MET*(Ex.15)
*MET*: c.3068_3082+6del	1	*MET*(Ex.13)::*MET*(Ex.15)
*MET*: c.3070_3082+22del	1	*MET*(Ex.13)::*MET*(Ex.15)
*MET*: c.3072_3082del	1	*MET*(Ex.13)::*MET*(Ex.15)
*MET*: c.3073_3082+25del	1	*MET*(Ex.13)::*MET*(Ex.15)
*MET*: c.3074_3082+4del	1	*MET*(Ex.13)::*MET*(Ex.15)
*MET*: c.3075_3082+2delinsGGA	1	*MET*(Ex.13)::*MET*(Ex.15)
*MET*: c.3075_3082+8del	1	*MET*(Ex.13)::*MET*(Ex.15)
*MET*: c.3076_3082+4del	2	*MET*(Ex.13)::*MET*(Ex.15)
*MET*: c.3076_3082+6del	1	*MET*(Ex.13)::*MET*(Ex.15)
*MET*: c.3078_3082+3del	1	*MET*(Ex.13)::*MET*(Ex.15)
*MET*: c.3079_3082+3del	1	*MET*(Ex.13)::*MET*(Ex.15)
*MET*: c.3079_3082+4del	1	*MET*(Ex.13)::*MET*(Ex.15)
*MET*: c.3080_3082+1del	1	*MET*(Ex.13)::*MET*(Ex.15)
*MET*: c.3080_3082+20delinsG	1	*MET*(Ex.13)::*MET*(Ex.15)
*MET*: c.3080_3082+5del	1	*MET*(Ex.13)::*MET*(Ex.15)
*MET*: c.3081_3082+11delinsCGC	1	*MET*(Ex.13)::*MET*(Ex.15)
*MET*: c.3081_3082+21del	1	*MET*(Ex.13)::*MET*(Ex.15)
*MET*: c.3081_3082+24del	1	*MET*(Ex.13)::*MET*(Ex.15)
***MET*: c.3081A>G p.Glu1027Glu**	1	*MET*(Ex.13)::*MET*(Ex.15)
*MET*: c.3082_3082+23del	1	*MET*(Ex.13)::*MET*(Ex.15)
*MET*: c.3082_3082+27del	1	*MET*(Ex.13)::*MET*(Ex.15)
*MET*: c.3082_3082+8del	1	*MET*(Ex.13)::*MET*(Ex.15)
*MET*: c.3082+1del	3	*MET*(Ex.13)::*MET*(Ex.15)
*MET*: c.3082+1G>A	25	*MET*(Ex.13)::*MET*(Ex.15)
*MET*: c.3082+1G>C	12	*MET*(Ex.13)::*MET*(Ex.15)
*MET*: c.3082+1G>T	18	*MET*(Ex.13)::*MET*(Ex.15)
*MET*: c.3082+2_3082+5delinsCATT	1	*MET*(Ex.13)::*MET*(Ex.15)
*MET*: c.3082+2T>A	3	*MET*(Ex.13)::*MET*(Ex.15)
*MET*: c.3082+2T>C	19	*MET*(Ex.13)::*MET*(Ex.15)
*MET*: c.3082+2T>G	1	*MET*(Ex.13)::*MET*(Ex.15)
*MET*: c.3082+3_3082+12del	1	*MET*(Ex.13)::*MET*(Ex.15)
*MET*: c.3082+3_3082+5del	1	*MET*(Ex.13)::*MET*(Ex.15)
*MET*: c.3082+3_3082+6del	1	*MET*(Ex.13)::*MET*(Ex.15)
*MET*: c.3082+3A>C	1	*MET*(Ex.13)::*MET*(Ex.15)
*MET*: c.3082+3A>G	17	*MET*(Ex.13)::*MET*(Ex.15)
*MET*: c.3082+3A>T	8	*MET*(Ex.13)::*MET*(Ex.15)
*MET*: c.3082-31_3082-25del	1	*MET*(Ex.13)::*MET*(Ex.15)
*MET*: c.3082del	1	*MET*(Ex.13)::*MET*(Ex.15)
*MET*: c.3082G>A p.Asp1028Asn	42	*MET*(Ex.13)::*MET*(Ex.15)
*MET*: c.3082G>C p.Asp1028His	38	*MET*(Ex.13)::*MET*(Ex.15)
*MET*: c.3082G>T p.Asp1028Tyr	13	*MET*(Ex.13)::*MET*(Ex.15)

*MET* exon 14 splice-site mutations were detected with a DNA-based NGS assay and verified by the RNA-based NGS assay. Two mutations were only detected with the RNA-based NGS assay at first, and one mutation was not reported on the DNA level (mutations in boldface).

c, cDNA level; Ex., exon; NGS, next-generation sequencing; No., recurrence of the mutation in the total cohort of 379 samples; p, protein level according to Human Genome Variation Society.

**Table 5:** MET Exon 14 Mutations That Do Not Lead to MET Exon 14 Skipping

Mutation detected by DNA-based assay	No.	RNA-based NGS	Co-occurring mutations
*MET*: c.2966A>G p.Tyr989Cys	1	Wild type	*EGFR*: c.2235_2249del p.Glu746_Ala750del; *TP53*: c.314G>A p.Gly105Asp; TP53: c.469G>T p.Val157Phe
*MET*: c.2968G>C p.Asp990His	1	Wild type	*TP53*: c.586C>T p.Arg196Ter
*MET*: c.3058G>C p.Asp1020His	1	Wild type	*FGFR1*: c.566G>T p.Arg189Leu; *KEAP1*: c.786C>G p.Phe262Leu; *KEAP1*: c.1408C>T p.Arg470Cys; *MAP2K1*: c.425G>T p.Cys142Phe; *TP53*: c.743G>T p.Arg248Leu
*MET*: c.3062A>G p.Tyr1021Cys	1	Wild type	*TP53*: c.596G>A p.Gly199Glu
*MET*: c.3062A>T p.Tyr1021Phe	1	Wild type	
*MET*: c.3064C>G p.Arg1022Gly	1	Wild type	*KEAP1*: c.1603G>T p.Glu535Ter; STK11: c.131_135del p.Lys44AsnfsTer117
*MET*: c.3082+11G>T	1	Wild type	*ERBB2*: c.2313_2324dup p.Tyr772_Ala775dup; TP53: c.692_694del p.Thr231del

No. is the recurrence of the mutation in the total cohort of 379 samples.

NGS, next-generation sequencing.

Deletions/insertions evaluated in this study were diverse. Of the 171 different *MET* exon 14 mutations in the cohort, 140 were different deletions/insertions. A total of 130 of the deletions/insertions were unique. They varied in size from a few nucleotides to a 63-bp deletion.

The most common mutations in the whole cohort were single-nucleotide variations at either the last nucleotide of *MET* exon 14 (c.3082G>X, NM_001127500; 24.6%; *https://www.ncbi.nlm.nih.gov/nuccore/NM_001127500*, last accessed May 6, 2026) or the first nucleotide in the intron behind *MET* exon 14 (c.3082+1G>X, NM_001127500; 14.6%; *https://www.ncbi.nlm.nih.gov/nuccore/NM_001127500*, last accessed May 6, 2026).

The RNA-based NGS assay was used in routine diagnostics as a complementary test for the detection of common fusion and exon skipping events in NSCLC samples and to confirm that the mutations present in *MET* exon 14 on the DNA level were leading to *MET* exon 14 skipping. Of the 171 different mutations in *MET* exon 14 and the splice sites of *MET* exon 14, 104 mutations were also analyzable with the RNA-based NGS assay and verified a *MET* exon 14 skipping mutation (Table 4). Two mutations were only detected with the RNA-based NGS assay at first, and one mutation was not reported on the DNA level.

**Figure 3:**
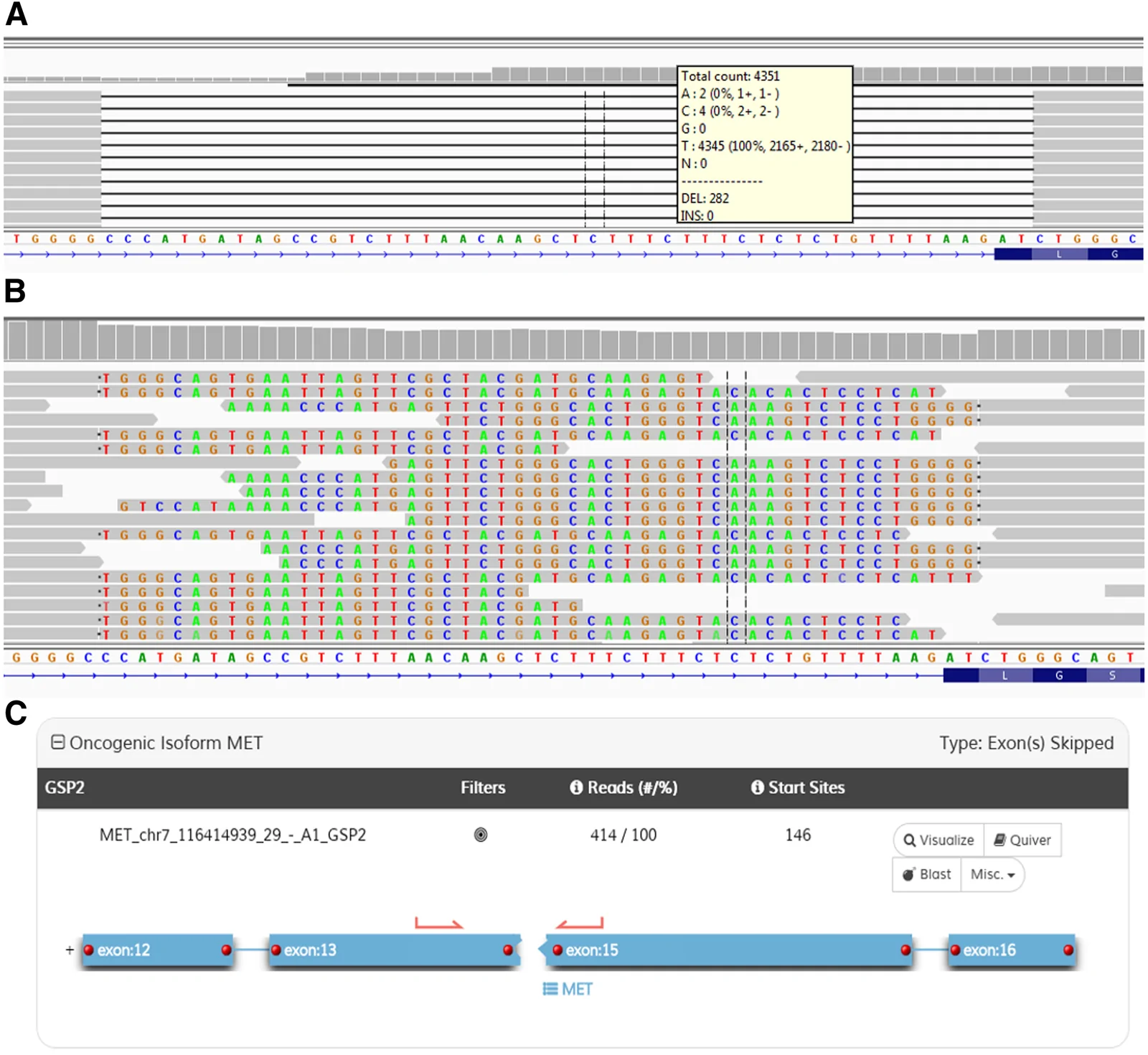
Sample with a large deletion in the splice site of *MET* exon 14, leading to *MET* exon 14 skipping. **A:***MET* exon 14 mutation c.2942-47_2944del detected with the DNA-based amplicon next-generation sequencing (NGS) panel visualized with the Integrative Genomics Viewer (IGV). **B:***MET* exon 14 mutation c.2942-47_2944del analyzed with the DNA-based hybridization NGS panel and not called in-house by the bioinformatic pipeline visualized with the IGV. **C:***MET*(exon 13)::*MET*(exon 15) fusion detected with the RNA-based NGS assay visualized with the Archer Analysis Software version 5.1.3.

**Figure 4:**
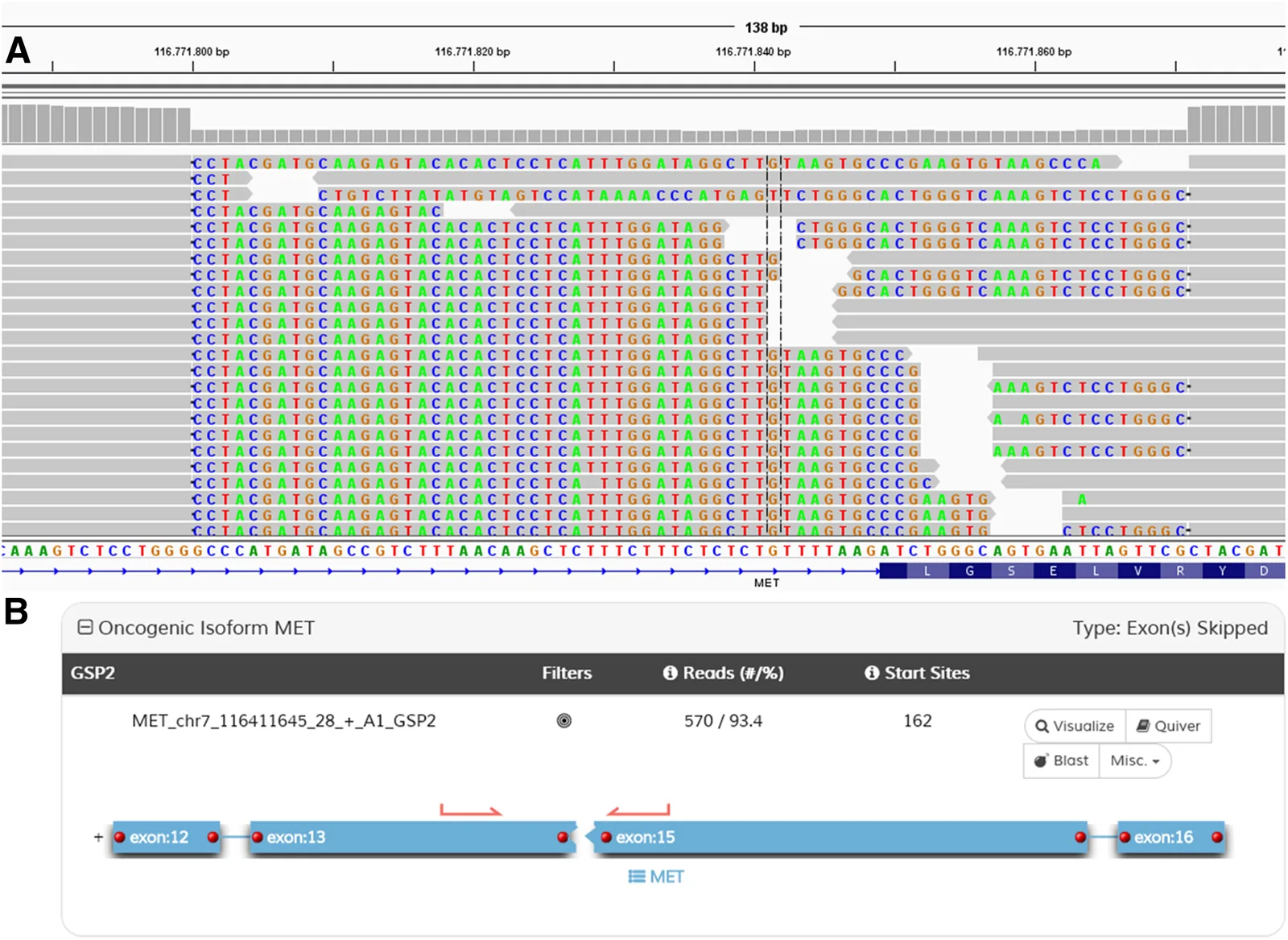
Sample with large deletion in the splice site of *MET* exon 14, leading to *MET* exon 14 skipping. **A:***MET* exon 14 mutation c.2942-49_2963delinsC analyzed with the DNA-based hybridization next-generation sequencing (NGS) panel and not called by the bioinformatic pipeline visualized with the Integrative Genomics Viewer. **B:***MET*(exon 13)::*MET*(exon 15) fusion detected with the RNA-based NGS assay visualized with the Archer Analysis Software version 6.2.7.

In three cases, the RNA-based assay led to a positive *MET* exon 14 skipping result, and the data output of two of the DNA-based hybridization NGS assays did not show an exon 14 mutation. When loading the binary alignment map (BAM) files in the Integrative Genomics Viewer (IGV),^28^ base substitutions were visible in the raw data of all three cases. In two cases, large deletions of 49 bp (c.2942-47_2944del) and 69 bp (c.2942-49_2963delinsC) were visible (Figures 3 and 4). These large deletions were not called with the implemented in-house pipeline using the caller mutect-2. The samples were then re-analyzed by structural variant caller Manta version 1.6.0^29^ and DELLY version 0.7.8^30^ for the detection of large deletions. In both cases, a large deletion was then detected by the structural variant caller. One of the samples (c.2942-47_2944del) was previously also analyzed by a DNA-based amplicon NGS assay, and the mutation was called on the DNA level (Figure 3). Interestingly, another large 49-bp deletion (c.2942-16_2976delinsG) was called with the in-house pipeline and the DNA-based hybridization NGS assay.

**Figure 5:**
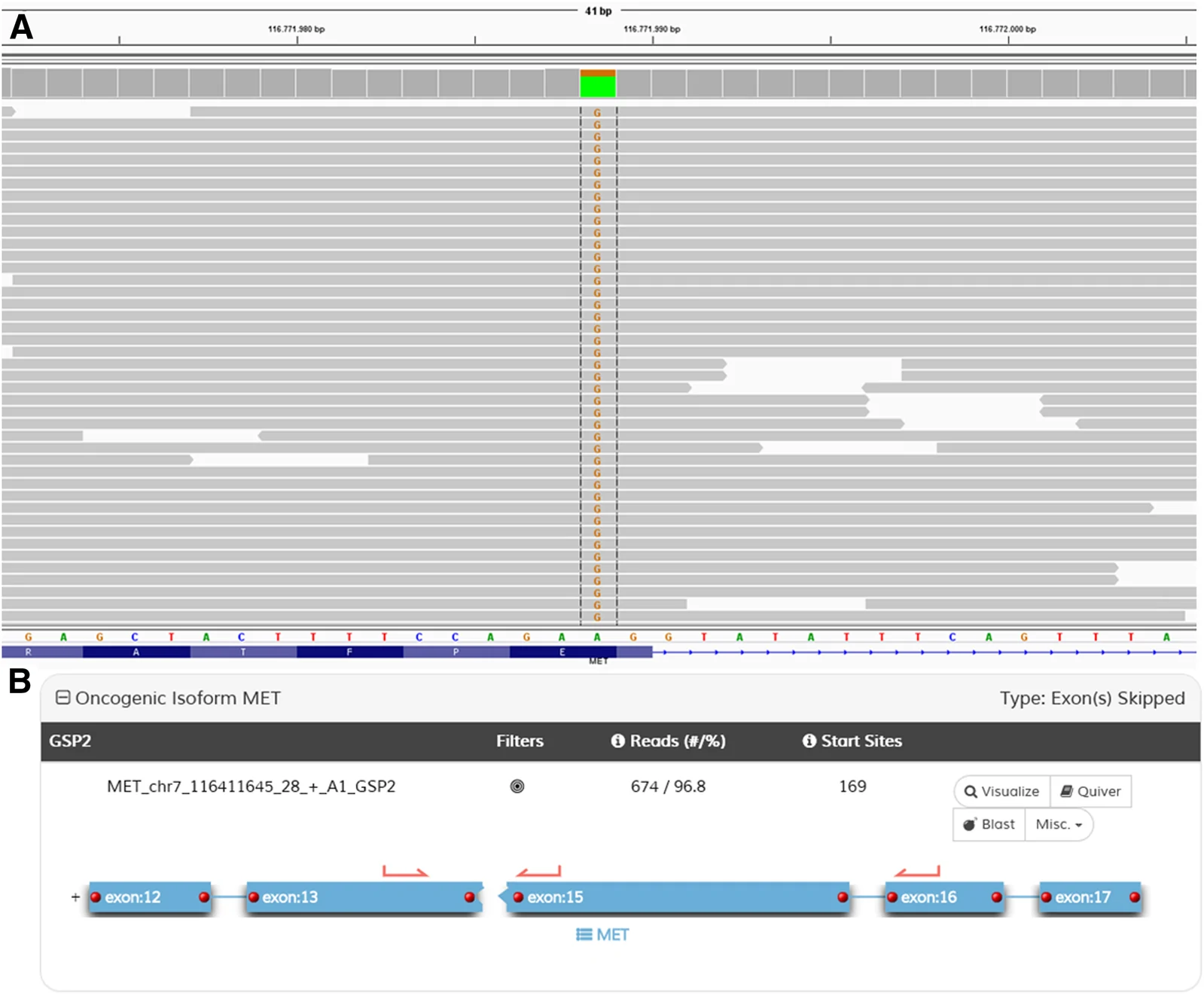
Sample with a synonymous point mutation in the splice site of *MET* exon 14, leading to *MET* exon 14 skipping. **A:***MET* exon 14 mutation c.3081A>G, p.Glu1027Glu detected with the DNA-based amplicon next-generation sequencing (NGS) panel visualized with the Integrative Genomics Viewer. **B:***MET*(exon 13)::*MET*(exon 15) fusion detected with the RNA-based NGS assay visualized with the Archer Analysis Software version 6.2.7.

In the third case, a synonymous mutation was visible in the mutation file and in the IGV [c.3081A>G, p.Glu1027Glu (NM_001127500, *https://www.ncbi.nlm.nih.gov/nuccore/NM_001127500*, last accessed May 6, 2026)] but was not reported at first. This mutation, however, was a base substitution in the splice site of *MET* exon 14, which led to *MET* exon 14 skipping on the RNA level (Figure 5).

Among the 379 *MET* exon 14–positive NSCLC samples, three samples had two *MET* exon 14 skipping variants each. One sample had two *MET* exon 14 skipping variants in *trans* configuration (c. [2942-18_2942-6del];[2942-16_2942-4del]), and the other two cases had one variant at the polypyrimidine tract (c.2942-32_2942-12del and c.2942-19_2949delinsCCTTT, respectively) and a frameshift in exon 14 (c.3005dup, p.Ser1003LysfsTer15) or a second variant at the splice donor site (c.3082G>C, p.Asp1028His) (Table 4). In these samples, it was not possible to assess the *trans* or *cis* configuration. In all three samples, *MET* exon 14 skipping was confirmed on the RNA level.

For seven variants, a suspected *MET* exon 14 skipping mutation was not confirmed on the RNA level. Six of these mutations were single-nucleotide variations in *MET* exon 14, and one variant was downstream of *MET* exon 14 (c.3082+11G>T) (Table 5). Two of these patient samples had driver variants in *EGFR* and *ERBB2* (*HER2*). Interestingly, another variant (c.2942-10C>A) 10 bases upstream of *MET* exon 14 led to *MET* exon 14 skipping (Table 4).

The remaining 57 variants in *MET* exon 14 or the splice sites of *MET* exon 14 could not be analyzed with the RNA-based NGS assay for a variety of reasons, either the tissue block and tNA extract were empty or the amount or quality of the RNA was too low to give any results. A total of 18 of the remaining 57 variants were confirmed as *MET* exon 14 skipping mutations by literature search. This left 39 unclassified mutations. Thirty of these were classical deletions/insertions in the splice sites of *MET* exon 14, two were truncating mutations within *MET* exon 14, six were single-nucleotide variations in exon 14, and one was a 1-bp duplication in intron 14 (11 nucleotides downstream of exon 14). A total of 26 of the 57 variants exhibited co-occurring mutations, predominantly in *TP53*. Notably, six variants were associated with established driver alterations in *KRAS* and *PIK3CA*. One *MET* variant (c.2942-42_2042-2del) co-occurred with a *KRAS* variant (p.Gly13Asp) and was classified by Awad et al^24^ as a *MET* exon 14 skipping variant (Supplemental Table S1).

### EQA Schemes for Molecular Testing of *MET* Exon 14 Skipping Organized by QuIP GmbH

#### EQA Sub-Scheme with FFPE Tissue

A total of 49 (98%) of the 50 participants were certified as having successfully participated in the first EQA sub-scheme for the assessment of *MET* exon 14 skipping mutations in FFPE tissue.

Sixteen participants received the first split, and four of the participants were unable to evaluate Case 7 (c.3082+1G>A). Material problems were excluded in this case, and no apparent pattern was observed. A total of 18 participants received the second split, and 1 participant was unable to evaluate Case 7 (wild type). No reasons were given. Sixteen participants received the third split, and only one participant was unable to evaluate Case 8 (C.3082+1G>A), and one participant misinterpreted Case 2 as false positive. To sum up, only one false positive result was detected, and problems were seen more frequently in missense mutations in intron 14 of *MET*.

**Figure 6:**
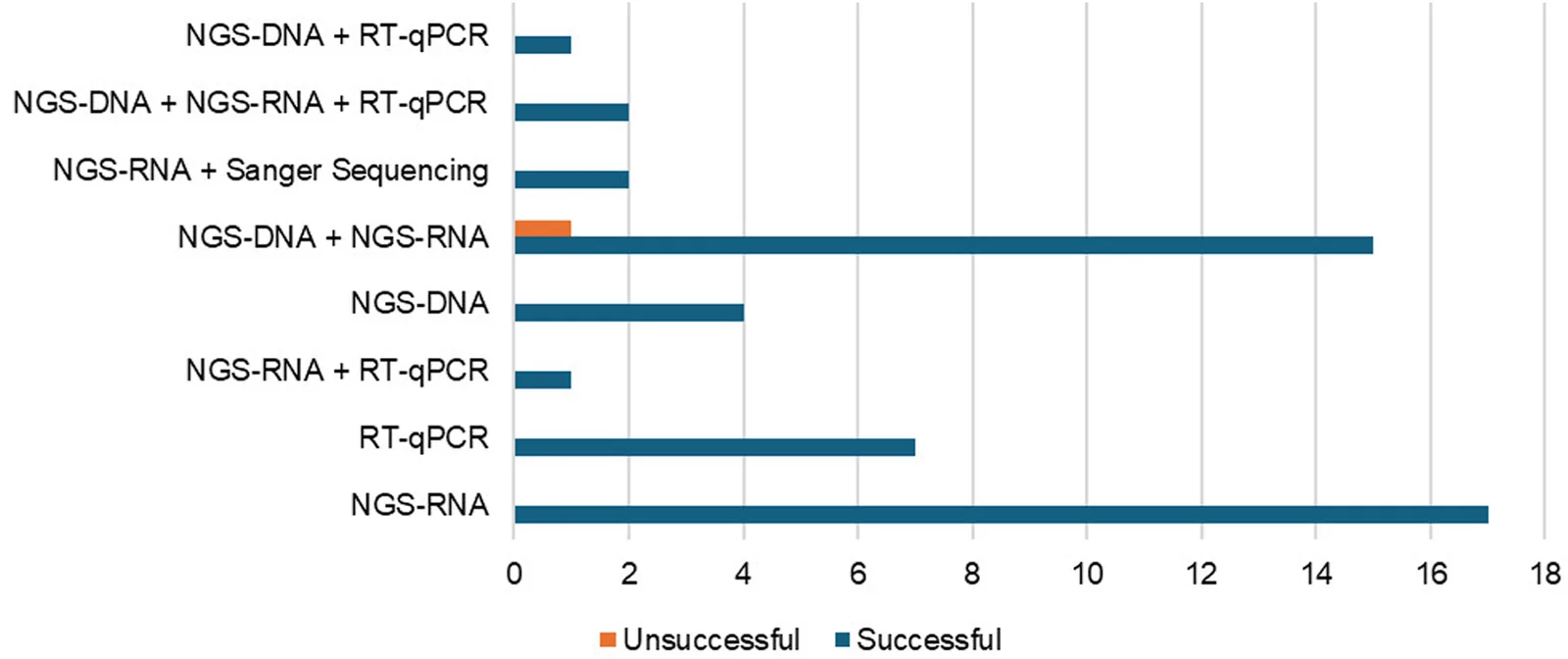
Outcomes of the external quality assessment sub-scheme with formalin-fixed, paraffin-embedded tissue depending on the methods used for the detection of *MET* exon 14 skipping mutations. Successful and unsuccessful participation were highlighted, along with the methods used. NGS, next-generation sequencing; RT-qPCR, quantitative RT-PCR.

The 50 participants used different molecular methods for the detection of *MET* exon 14 skipping mutations (Figure 6). Four participants used DNA only, 25 participants used RNA only, and 21 participants used DNA- and RNA-based methods in parallel. DNA- and/or RNA-based NGS assays were most commonly used. On the DNA level, mainly kits from Qiagen and Thermo Fisher Scientific were used (Supplemental Table S2). On the RNA level, 30% of the participants used different Archer (IDT) and Thermo Fisher Scientific assays (Supplemental Table S3). Two participants used Sanger sequencing for DNA-based detection, and eight participants used quantitative RT-PCR for RNA-based detection. Here, the assay from Biocartis was used most frequently. The only institute that failed the EQA sub-scheme used DNA- and RNA-based NGS.

### EQA Sub-Scheme with Liquid Biopsy Samples in 2022

**Figure 7:**
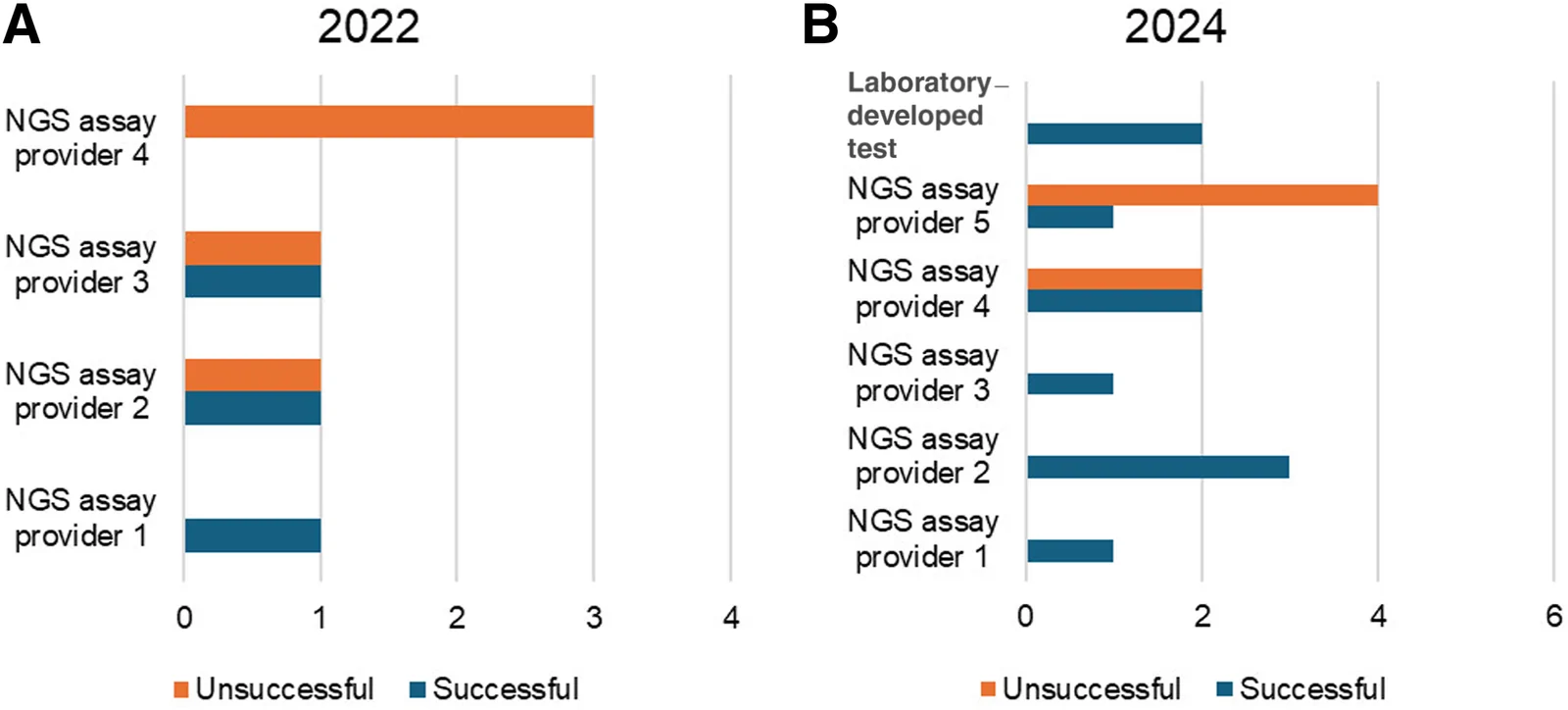
Outcomes of the external quality assessment (EQA) sub-scheme with liquid biopsy samples for the detection of *MET* exon 14 skipping mutations on the DNA level. All participants used DNA-based next-generation sequencing (NGS) methods. Successful and unsuccessful participation were highlighted, along with the methods used. **A:** Outcome of the EQA sub-scheme with liquid biopsy samples in 2022. **B:** Outcome of the EQA sub-scheme with liquid biopsy samples in 2024.

In the EQA sub-scheme conducted in 2022, only three of eight participants received certificates of successful participation (3/8; 37.5%) (Figure 7A). All participants used DNA-based NGS assays for the detection of mutations in liquid biopsy samples. The participants used different panels from four different providers (Figure 7A and Supplemental Table S4).

A total of 25% (20/80) of all submitted results were false negative, and only one participant reported for Case 9 an additional mutation, which was not included in the test set. Detection problems seemed to be associated either with insufficient sensitivity of methods used or data analysis. Only one participant submitted a wrong annotation of one mutation (c.2942-18_2942-8del instead of c.2942-18_2942-5del); however, the correct annotation was not mandatory for successful participation. In all other cases, the annotation was given correctly, when the mutation was successfully detected. A more detailed error analysis was performed in the EQA sub-scheme with liquid biopsy samples in 2024 and is highlighted in the next paragraph.

### EQA Sub-Scheme with Liquid Biopsy Samples in 2024

**Figure 8:**
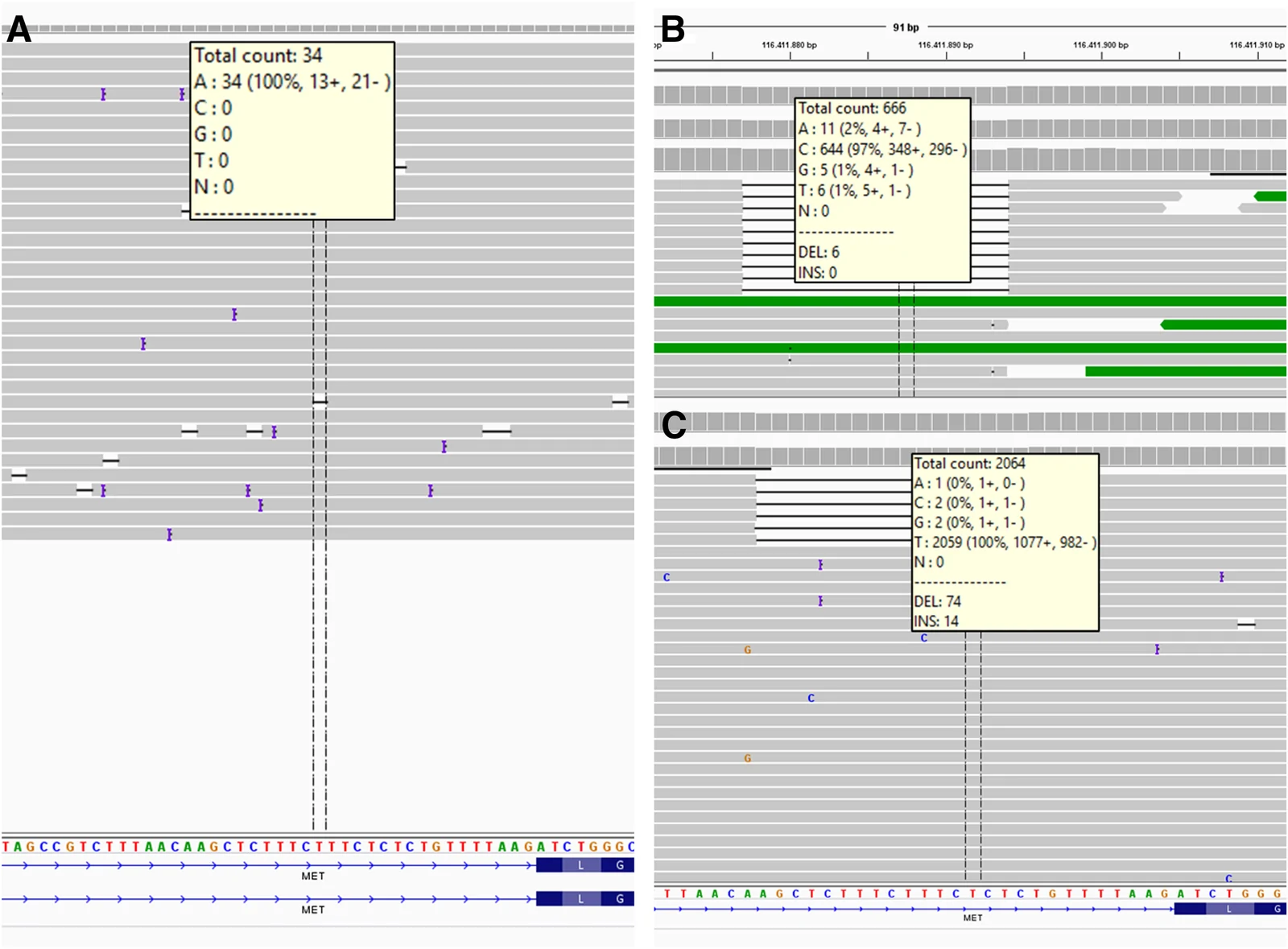
Results of Case 1 (c.2942-3_2942-10del) of the external quality assessment sub-scheme for *MET* exon 14 skipping with liquid biopsy samples (2024). **A:** False-negative results because of coverage of only 34×. **B:** False-negative results because of variant calling. Mutation is present in the Integrative Genomics Viewer (IGV) with an allelic fraction of 0.89% and a total coverage of 666×. Mutation was not called by the bioinformatics pipeline. **C:** False-negative results because of bioinformatics problems. Mutation is present in the IGV with an allelic fraction of 3.46% and a total coverage of 2064×. Mutation was not called by the bioinformatics pipeline.

In the EQA sub-scheme conducted in 2024, 10 of 16 participants received certificates of successful participation (10/16; 63%) (Figure 7B and Supplemental Table S5). Although 63% of the participants determined the mutation status of each case correctly, results deviating from the reference values were submitted for each of the 10 cases. The six participants who were unsuccessful had problems analyzing the *MET* exon 14 skipping status in several cases and reported false-negative results in three or more cases. The respective participants were asked to check the raw data for the cases they indicated as false negative and were asked to review their bioinformatics pipelines. Raw data were also analyzed by the lead panel institute using the IGV. Here, two main problems were identified: first, an insufficient coverage of the target region; and second, the detection threshold of the bioinformatic pipelines. Case 1, harboring the *MET* exon 14 skipping mutation c.2942-26_2942-10del with 2% allelic fraction, was the prime example for the reasons causing false-negative results. This was a 16-bp deletion in intron 13 of *MET*. One participant with false-negative results had a coverage of only 34× at this specific position (Figure 8A). This was not sufficient to detect variants with a mutant allelic fraction of 2%. Another participant had a coverage of 666× at this position, but the bioinformatics pipeline did not call this variant. After analyzing the raw data, the variant could be detected with a mutant allelic fraction of 0.89% in the IGV (Figure 8B). In this case, the variant was filtered out by the bioinformatics pipeline because of the low allelic fraction. This could also be observed by a third participant with a coverage of 2064× but a false-negative result in the EQA sub-scheme. The raw data showed that the mutation was present with an allelic fraction of 3.46% (Figure 8C). This demonstrated that not only the wet-laboratory part of method but also the bioinformatics pipeline should be validated carefully. To exclude false-negative results, raw data should be checked manually during method validation. In addition, the pre-analytics may have an influence on the success of the mutation detection. In Case 6 with a deletion in intron 13 (c.2942-18_2942-5del), one participant reported a false-negative result. Checking the raw data, the coverage was 686×, and no mutation could be seen. The expected mutant allelic fraction was 2.06%; therefore, reads harboring the deletion should be visible in the raw data. Here, technical problems during the pre-analytic phase seemed to have occurred. In addition, participants were asked for precise information about the annotation of the detected mutations according to standard HGVS nomenclature and the allelic fraction of the mutation. The annotation at the c-DNA level was correctly stated by most participants, who correctly classified the case as positive. Only in Cases 3 and 6, both containing the *MET* exon 14 skipping mutation c.2942-18_2942-5del, three to four participants failed to name the mutation correctly, respectively.

To sum up, *MET* exon 14 skipping in FFPE tissue can be reliably detected in nearly all participating institutes. Nearly all samples were correctly detected regardless of localization or type of variant (missense mutation, deletions, or deletions/insertions). No superior manufacturer could be detected; however, DNA- and RNA-based NGS should be combined. For the liquid biopsy part, an improvement in the detection of *MET* exon 14 skipping mutations can be seen from 2022 to 2024; however, more improvements are needed concerning the detection of low allelic fraction variants (sensitivity and coverage) as well as the bioinformatics tools (variant calling). Here again, a thoroughly validated NGS method should be the method of choice.

## Discussion

The large, growing number of detected *MET* alterations in NSCLC has shown the important role of the encoded receptor tyrosine kinase for targeted therapy approaches. Therefore, routine testing should be standard of care for patients with NSCLC. This is especially because the US Food and Drug Administration and European Medicines Agency approved MET inhibitors for advanced NSCLC with *MET* exon 14 skipping mutations.

In this study, a large cohort of 379 NSCLCs with *MET* exon 14 mutations was assessed. The 379 NSCLC samples harbored 171 different *MET* exon 14 and *MET* exon 14 splice-site mutations, showing the variety of these mutations, which is in accordance with previous studies.^24^^,^^27^^,^^31^

In this study, 114 samples carrying 1 of the 171 detected variants were analyzed with both a DNA- and an RNA-based NGS assay. In 107 of the 114 variants, *MET* exon 14 skipping was verified with the RNA-based NGS assay as only the splicing effect qualifies for targeted therapy.^1^^,^^32^ Seven samples showing single-nucleotide variations in the *MET* exon 14 region were analyzed as wild type with the RNA-based NGS assay, thus not leading to *MET* exon 14 skipping. Six of these variants were single-nucleotide variations in exon 14, and one was a single-nucleotide variation in intron 14 of *MET* (c.3082+11G>T). Interestingly, another single-nucleotide variant in intron 13, 10 bases before *MET* exon 14 (c.2942-10C>A), did lead to *MET* exon 14 skipping in the RNA-based NGS. Thus, it seems that the variant c.2942-10C>A is still part of the splice site of *MET* exon 14 and the variant c.3082+11G>T is not in the splice site of *MET* exon 14. This highlights the need for verification of both *MET* exon 14 splice sites using RNA-based NGS. The remaining 57 variants in *MET* exon 14 or the splice sites of *MET* exon 14 detected in this study could not be analyzed with the RNA-based NGS assay because either no tumor material was left or the RNA quality was not sufficient. However, 18 variants were already described in the published literature as *MET* exon 14 skipping mutation.^4^^,^^7^^,^^24^, ^25^, ^26^, ^27^ Here, inconsistent nomenclature of somatic variants and two different transcripts used in the literature are of concern. In the literature and in online databases, two different mRNA transcripts are frequently used for the *MET* gene [transcript NM_000245 (*https://www.ncbi.nlm.nih.gov/nuccore/NM_000245*, last accessed May 6, 2026) and transcript NM_001127500 (*https://www.ncbi.nlm.nih.gov/nuccore/NM_001127500*, last accessed May 6, 2026)]. In transcript NM_000245 (*https://www.ncbi.nlm.nih.gov/nuccore/NM_000245*, last accessed May 6, 2026), the splice site of exon 14 is located at codon 1010, and in transcript NM_001127500 (*https://www.ncbi.nlm.nih.gov/nuccore/NM_001127500*, last accessed May 6, 2026), a rare polymorphism at codon 1010 (T1010I) is reported; this can lead to a misconception of the detected mutation in the clinic.^15^^,^^33^ Furthermore, strict adherence to HGVS nomenclature for variant annotation and reporting is critical, as it is the only way to ensure accurate and meaningful comparison of variants across databases and the scientific literature.

In the present study, 39 of the 171 detected variants remained unclassified. However, 30 of these were classical deletions/insertions affecting splice donor or acceptor site and/or the polypyrimidine tract, probably resulting in *MET* exon 14 skipping, as previously described.^31^

Because of the great diversity of *MET* exon 14 skipping mutations, it is particularly important to choose a detection method that covers all possible *MET* exon 14 splice site mutations. DNA- and RNA-based NGS approaches have shown the highest success. However, in DNA-based assays, intronic regions have to be covered sufficiently to be able to detect large deletions/insertions in these regions. Other studies showed that *MET* exon 14 skipping alterations can be missed by amplicon-based sequencing if the assay is not optimized for this purpose.^8^^,^^9^^,^^32^^,^^34^ In the present study, it was shown that also DNA-based hybridization NGS approaches and bioinformatics pipelines might fail to detect *MET* exon 14 skipping mutations. Therefore, a profound validation of the analysis itself as well as the bioinformatics pipeline is essential, and routine diagnostics should be performed under high-quality standards. Analyzing DNA- and RNA-based approaches in parallel has the advantage that you are less likely to miss a *MET* exon 14 skipping mutation. The bioinformatics pipeline for the DNA-based hybridization assay failed to detect two large deletions of 49 and 69 bp in length at first. After positive results revealed by the RNA-based NGS assay, the DNA-based data was re-evaluated. The deletions were visible in the IGV but were not called by mutect 2. For the detection of deletions in the above-mentioned range in hybridization-based data, a structural variant caller like Manta/Delly has to be applied. Interestingly, the 49-bp deletion was also analyzed with a DNA-based amplicon NGS assay and here the deletion was called by the accompanying pipeline. This DNA-based amplicon assay was optimized for large *MET* exon 14 skipping deletions by more overlapping amplicons and primer pairs. If assays are not optimized for the detection of *MET* exon 14 skipping mutations and primer pairs are located at the position of the deletion or the coverage is not sufficient enough in intronic regions surrounding *MET* exon 14, only wild-type sequences would be called.^8^^,^^32^^,^^34^ Unfortunately, the chemistry and reagents for this amplicon-based method were discontinued by the supplier, and this study was not able to test and use this approach any further. Overall, the simultaneous use of DNA- and RNA-based NGS seems to be the method of choice. However, because of small biopsies and the limited material availability, this is not always possible.

It was also shown that synonymous variants, like p.Glu1027Glu (NM_001127500, *https://www.ncbi.nlm.nih.gov/nuccore/NM_001127500*, last accessed May 6, 2026) in the splice sites of *MET* exon 14 can lead to exon skipping. Because such synonymous variants are often filtered out by the bioinformatics pipeline of a DNA-based assay, *MET* exon 14 skipping can easily be missed and would only be detected using an additional RNA-based approach. Thus, bioinformatics pipelines should be adjusted to leave synonymous splice variants in the exon/intron border. This is especially crucial when commercial software is not optimized for these rare variants.^35^

RNA-based assays, however, can be hampered by poor RNA integrity, and RNA quality should be closely monitored when using FFPE material.^11^ Overall, the present study has shown that the RNA-based NGS assay can be used to determine whether a specific *MET* splice-site mutation results in altered splicing and the loss of exon 14. It was shown that with the RNA-based NGS assay, little tNA is needed to achieve reliable results. In the study, the determined limit of detection of the RNA-based NGS assay was 10-ng tumor tNA, representing a tumor cell content of 10% and an allelic fraction of 5% if 200-ng tNA was used in the assay. This is in concordance with the published literature.^11^^,^^36^ Thus, the careful selection of a reliable NGS assay and validation of technical sensitivity are of utmost importance for routine diagnostics.

In DNA-based approaches, the splicing effect can only be substantiated by literature data. Nevertheless, with a DNA-based approach, the exact mutation can be named, which is not possible at the RNA level. Ideally, a DNA and RNA approach would be used simultaneously, but this is often not possible when using small lung cancer biopsies.^8^^,^^34^

Nonetheless, there is still the need for further development of sensitive and quality assured molecular testing methods, especially under the requirements of the new In Vitro Diagnostics Regulation. This is especially important in Europe, as laboratories are free to choose the testing method for the detection of *MET* exon 14 skipping mutations. Therefore, the first multinational EQA schemes were conducted together with the QuIP GmbH for the assessment of *MET* exon 14 skipping mutations in FFPE tissue and liquid biopsy samples. Fifty institutes from Germany, Austria, and Switzerland registered for the EQA sub-scheme with FFPE material and submitted their results.

In total, 49 of 50 institutes (98%) successfully participated in the EQA sub-scheme, which is an excellent pass rate. EQA sub-scheme participants were able to freely choose the methods for the detection of *MET* exon 14 skipping mutations. Most participants used NGS, but RT-PCR–based methods and Sanger sequencing were also used. A variety of different reagent systems and evaluation pipelines led to the correct results. Only one case was rated as a false positive by one participant using DNA- and RNA-based NGS. The results of the various participating institutions showed that the detection of *MET* exon 14 skipping mutations is already well established on FFPE tissue in the participating countries.

In the first EQA sub-schemes for *MET* exon 14 skipping with liquid biopsy samples, a significant improvement in pass rates was observed between 2022 and 2024, increasing from 37.5% to 63%. Despite this progress, the reliability of results in liquid biopsy testing remains insufficient, with major problems occurring in cases with low allelic fractions or intronic deletions, which led to false-negative results because of limited assay sensitivity or bioinformatics failure to detect larger intronic deletions. No case was correctly identified by all participants. The largest factor identified was insufficient sequencing coverage. A key factor contributing to frequent false-negative results in cases with low allelic fractions or intronic deletions is not only the sequencing depth, but also the bioinformatic filter settings. For reliable detection of variants at approximately 1% mutant allele fraction, targeted NGS assays generally aim for a sequencing coverage of ≥1000× unique reads, as this depth improves sensitivity for low-frequency variant calls in clinical sequencing workflows.^37^^,^^38^ Additionally, filter parameters have to be carefully optimized to ensure that low-frequency and intronic variants are not missed.

Participants who achieved a low coverage of <100× were not able to detect the *MET* exon 14 skipping mutation. Regarding the bioinformatics pipeline, mutations were visible in the raw reads but were not called because of a high detection threshold or wrong parameters in the pipeline. This showed that not only the wet-laboratory procedures but also the bioinformatic data analysis pipelines require careful selection and validation.^10^ This is even more important in the analysis of liquid biopsy samples as the blood might be hemolytic and the fraction of circulating tumor DNA released by cancer cells in the bloodstream might be low in samples of patients with NSCLC.^39^^,^^40^

In conclusion, the increasing recognition of *MET* alterations, particularly *MET* exon 14 skipping mutations in NSCLC, emphasizes the need for standardized testing under high quality control in clinical practice. The great variety of identified mutations underlines the importance of using comprehensive detection methods. Although the EQA sub-scheme with FFPE tissues samples is promising, further improvements are needed for the analysis of liquid biopsy samples that necessitate further methodological refinement.

Looking ahead, advancements in cell-free RNA analysis hold great potential for improving the detection of *MET* exon 14 skipping mutations in the clinic. Enhanced extraction techniques, sequencing technologies, and bioinformatics will be crucial for increasing sensitivity and specificity.^41^ Integrating cell-free RNA analysis into routine diagnostics could enable timely therapeutic interventions. Addressing current bioinformatics limitations and ensuring adequate coverage for liquid biopsies will be essential for future mutation detection.

## Disclosure Statement

C.H. has received honoraria from Novartis; and travel costs from Novartis. M.A.I. has received honoraria from AstraZeneca, Bristol Meyers Squibb (BMS), Novartis, Merck, and GSK. K.I. had been on advisory boards from GSK, Amgen GmbH, and Merck Sharp & Dohme (MSD); has received honoraria from the University Hospital Cologne; and was a former employee of Quality in Pathology (QuIP) GmbH. J.S.-H. has received honoraria from AstraZeneca, Molecular Health, Targos, Biocartis, QuIP/GSK, and Novartis; and travel costs from Novartis and AstraZeneca. S.W.-R. has received honoraria from AstraZeneca; and travel costs from AstraZeneca. C.J. has received honoraria from Menarini, Novartis, and QuIP. J.F. has received honoraria from Menarini. R.R. has received honoraria from Janssen-Cilag and AstraZeneca. A.M.S. has been on the advisory board from BeiGene, Pierre Fabre Oncology, AstraZeneca, MSD, and BMS; and has received travel costs from AstraZeneca and BMS. S.M.-B. has received honoraria/been on the advisory board from AstraZeneca, Novartis, Roche, BMS, Illumina, DLS, Daiichi-Sankyo, QuIP, and Stemline; and has received third-party funding from AstraZeneca, Roche, Zytovision, and QuIP. All other authors declare no financial or nonfinancial competing interests.
