# Supplementary material for: Pitfalls in Detecting MET Exon 14 Skipping Variants by DNA- and RNA-Based Next-Generation Sequencing Technologies in a Large Real-World Cohort and Results of the First Multinational External Quality Assessment Schemes
Source: J Mol Diagn. 2026 Jun 4;28(8):625–41. doi: 10.1016/j.jmoldx.2026.05.007 (PMC13494073; doi:10.1016/j.jmoldx.2026.05.007)
Supplement: Supplementary file 1 [file mmc1.docx]

**Supplemental Table S1: 57 remaining mutations in the splice site of *MET* exon 14 that could not be analyzed with the RNA-based NGS assay.** 18 mutations were confirmed as exon 14 skipping mutations by the literature.

| **Mutation detected by DNA-based NGS assay** | **No.** | **Literature** | **Co-occuring mutations** |
| --- | --- | --- | --- |
| MET: c.2942-14_2945del | 1 | Frampton et al, Cancer Discovery 2015 [5] | *TP53*: c.404G>T p.Cys135Phe |
| MET: c.2942-15_2948del | 1 | Frampton et al, Cancer Discovery 2015 [5] |  |
| MET: c.2942-16_2975delins36bp | 1 | Awad et al., J Clin Oncol 2016 [24] |  |
| MET: c.2942-17_2942-7delinsC | 1 |  |  |
| MET: c.2942-18_2942-2del | 1 | Tong et al., Clin Cancer Res 2016 [25] | *PIK3CA*: c.3140A>G p.His1047Arg |
| MET: c.2942-19_2942-3del | 1 | Awad et al., J Clin Oncol 2016 [24] |  |
| MET: c.2942-20_2944del | 1 | Onozato et al J Thorac Oncol 2009 [7] |  |
| MET: c.2942-22_2942-3delinsAAAC | 1 |  |  |
| MET: c.2942-22_2942-9del | 1 | Tong et al., Clin Cancer Res 2016 [25] |  |
| MET: c.2942-22_2953del | 1 |  |  |
| MET: c.2942-23_2949del | 1 | Onozato et al J Thorac Oncol 2009 [7] | *TP53*: c.538G>T p.Glu180Ter |
| MET: c.2942-24_2942-11delinsTC | 1 |  |  |
| MET: c.2942-27_2942-15del | 1 |  | *TP53*: c.377A>G p.Tyr126Cys |
| MET: c.2942-27_2942-16del | 1 |  | *KRAS*: c.35G>T p.Gly12Val; *TP53*: c.742C>T p.Arg248Trp |
| MET: c.2942-28_2942-9del | 1 |  |  |
| MET: c.2942-32_2942-9del | 1 | Frampton et al., Cancer Discov 2015 [5] | *TP53*: c.796G>A p.Gly266Arg; |
| MET: c.2942-42_2042-2del | 1 | Awad et al., J Clin Oncol 2016 [24] | *KRAS*: c.38G>A p.Gly13Asp; ; *TP53*: c.731G>T p.Gly244Val |
| MET: c.2942-47_2942-21del | 1 |  |  |
| MET: c.2942-6_2942-1del | 1 |  |  |
| MET: c.2944C>G p.Leu982Val | 1 |  | *KEAP1*: c.755_756delinsTT p.Trp252Phe; ; *TP53:* c.811G>T p.Glu271Ter |
| MET: c.2947G>C p.Gly983Arg | 1 |  | *TP53*: c.610G>T p.Glu204Ter |
| MET: c.2951G>T p.Ser984Ile | 2 |  | *TP53*: c.454C>T p.Pro152Ser |
| MET: c.2996_3003del p.Asp999GlyfsTer16 | 1 |  |  |
| MET: c.3049G>T p.Glu1017Ter | 1 |  | *KRAS*: c.35G>A p.Gly12Asp |
| MET: c.3055_3063del p.Val1019_Tyr1021del | 1 | Kong-Beltran et al., 2006, Cancer Res [26] |  |
| MET: c.3056_3061del p.Val1019_Tyr1021delinsD | 1 | Schrock et al., JTO 2016 [27] |  |
| MET: c.3059_3082+8del | 1 | Frampton et al, Cancer Discovery, 2015; Awad et al, J Clin Oncol, 2016 [5,24] |  |
| MET: c.3061_3063del p.Tyr1021del | 1 | Schrock et al., JTO 2016 [27] |  |
| MET: c.3063_3082+22del | 1 |  |  |
| MET: c.3065_3082+9del | 1 |  |  |
| MET: c.3069_3082del p.Thr1024SerfsTer4 | 1 |  |  |
| MET: c.3070_3082+7del | 1 |  | *TP53*: c.677_686del p.Gly226ValfsTer18 |
| MET: c.3071_3082+2del | 1 | Onozato et al J Thorac Oncol 2009 [7] |  |
| MET: c.3072_3081del p.Phe1025IlefsTer19 | 1 |  | *TP53*: c.713G>T p.Cys238Phe |
| MET: c.3072_3082+4del | 2 |  |  |
| MET: c.3073_3082del p.Phe1025IlefsTer19 | 1 |  |  |
| MET: c.3074_3082+5delinsATC | 1 |  |  |
| MET: c.3075_3082del p.Phe1025LeufsTer5 | 1 |  | *NRAS*: c.190T>G p.Tyr64Asp; *TP53*: c.731G>A p.Gly244Asp |
| MET: c.3077_3082+10del | 1 |  | *TP53*: c.224delC p.Pro75LeufsTer48 |
| MET: c.3079_3082+5del | 1 |  |  |
| MET: c.3081_3082+10del | 1 | Frampton et al, Cancer Discovery, 2015 [5] | *TP53*: c.853G>A p.Glu285Lys |
| MET: c.3081_3082+15del | 1 |  |  |
| MET: c.3082_3082+16delinsA | 1 |  |  |
| MET: c.3082_3082+1delinsTT | 1 |  | *TP53*: c.548C>G p.Ser183Ter |
| MET: c.3082+1_3082+6del | 1 |  |  |
| MET: c.3082+11dup | 1 |  | *TP53*: c.701A>G p.Tyr234Cys |
| MET: c.2942-12_2942-9del | 1 |  |  |
| MET: c.2942-15_2942-9delinsA | 1 |  | *KRAS*: c.35G>A p.Gly12Asp; *TP53*: c.714_725del p.Asn239_Cys242del |
| MET: c.2942-1G>T | 3 |  | *TP53*: c.839G>A p.Arg280Lys |
| MET: c.2942-26_2942-15del | 1 |  | *TP53*: c.843C>A p.Asp281Glu |
| MET: c.3001C>A p.Leu1001Ile | 1 |  | *NTRK1*: c.2105G>A p.Arg702His; *TP53*: c.508_521del p.Thr170AlafsTer6 |
| MET: c.3010_3014delinsT p.Ala1004Ter | 1 |  | *PIK3CA*: c.1624G>A p.Glu542Lys; *TP53*: c.536A>G p.His179Arg |
| MET: c.3025C>T p.Pro1009Ser | 1 |  | *TP53*: c.638G>T p.Arg213Leu |
| MET: c.3070A>G p.The1024Ala | 1 |  | *TP53*: c.488A>G p.Tyr163Cys |
| MET: c.3071_3082del p.The1024_Asp1028delinsAsn | 1 |  | *TP53*: c.853G>A p.Glu285Lys |
| MET: c.2942-28_2942-1del | 1 | Onozato et al J Thorac Oncol 2009 [7] |  |
| MET: c.2942-9_2943delinsAAAA | 1 | Onozato et al J Thorac Oncol 2009 [7] |  |

No.: Recurrence of the mutation in the total cohort of 379 samples.
