# Supplementary material for: Pitfalls in Detecting MET Exon 14 Skipping Variants by DNA- and RNA-Based Next-Generation Sequencing Technologies in a Large Real-World Cohort and Results of the First Multinational External Quality Assessment Schemes
Source: J Mol Diagn. 2026 Jun 4;28(8):625–41. doi: 10.1016/j.jmoldx.2026.05.007 (PMC13494073; doi:10.1016/j.jmoldx.2026.05.007)
Supplement: Supplementary file 2 [file mmc2.docx]

**Supplemental Table S2: DNA-based methods used and results of the EQA sub-scheme with FFPE tissue.**

| **Manufacturer** | **Assay used** | **No. of participants** | **Success rate (%)** |
| --- | --- | --- | --- |
|  | no DNA-based methods used | 25 | 25 (100) |
| Agilent | SureSelect XT HS2 Lung Assay | 1 | 1 (100) |
| AmoyDx | AmoyDx HANDLE Classic NGS Panel | 1 | 1 (100) |
| ArcherDx | Archer VariantPlex nNGM-Panel v2 | 1 | 1 (100) |
| Illumina | AmpliSeq for Illumina Focus Panel | 2 | 2 (100) |
|  | TruSight Oncology 500 | 1 | 1 (100) |
| Qiagen | GeneReader Lung DNA FFPE Panel | 1 | 0 (0) |
|  | QIAseq multimodal Lung Panel | 2 | 2 (100) |
|  | QIAseq Custom Targeted DNA nNGM-Panel v2 | 3 | 3 (100) |
|  | QIAseq Custom Targeted DNA nNGM-Panel v2.1 | 1 | 1 (100) |
|  | QIAseq Custom Targeted DNA nNGM-Panel v1 | 1 | 1 (100) |
|  | QIAseq Targeted DNA Custom Panel | 2 | 2 (100) |
| Thermo Fisher | Oncomine Comprehensive Assay v3 | 2 | 2 (100) |
|  | Andere: Oncomine Precision Assay Gx | 1 | 1 (100) |
|  | nNGM-Panel v2 | 1 | 1 (100) |
| Illumina + Thermo Fisher | nNGM-Panel v2 + nNGM-Panel v2 | 1 | 1 (100) |
| unknown | nNGM-Panel v2 | 2 | 2 (100) |
|  | Sanger sequencing | 2 | 2 (100) |

nNGM: National Netwerk Genomic Medicine. %: Success rate for each method used. No: Number
