# Supplementary material for: Pitfalls in Detecting MET Exon 14 Skipping Variants by DNA- and RNA-Based Next-Generation Sequencing Technologies in a Large Real-World Cohort and Results of the First Multinational External Quality Assessment Schemes
Source: J Mol Diagn. 2026 Jun 4;28(8):625–41. doi: 10.1016/j.jmoldx.2026.05.007 (PMC13494073; doi:10.1016/j.jmoldx.2026.05.007)
Supplement: Supplementary file 3 [file mmc3.docx]

**Supplemental Table S3: RNA-based methods used and results of EQA sub-scheme with FFPE tissue.**

| **Method** | **Assay used** | **No. of participants** | **Success rate (%)** |
| --- | --- | --- | --- |
|  | no RNA-based method used | 4 | 4 (100) |
| NGS | AmoyDx HANDLE Classic NGS Panel (AmoyDx) | 1 | 1 (100) |
|  | Archer Fusion Plex Lung (ArcherDx) | 11 | 11 (100) |
|  | Archer FusionPlex Comprehensive Thyroid & Lung (CTL) Kit (ArcherDx) | 3 | 3 (100) |
|  | AmpliSeq for Illumina Focus Panel (Illumina) | 4 | 4 (100) |
|  | TruSight Tumor 170 (Illumina) | 1 | 1 (100) |
|  | QIAact Lung RNA UMI Panel (Qiagen) | 1 | 1 (100) |
|  | QIAseq multimodal lung cancer panel (Qiagen) | 2 | 2 (100) |
|  | QIAGEN QIAseq Targeted RNAscan Panel (Qiagen) | 2 | 2 (100) |
|  | GeneRead Lung Fusion Panel 2.0 (Qiagen) | 1 | 0 (0) |
|  | Oncomine Precision Assay (Thermo Fisher) | 1 | 1 (100) |
|  | Oncomine Precision Assay Gx (Thermo Fisher) | 1 | 1 (100) |
|  | Oncomine Focus Assay (Thermo Fisher) | 6 | 6 (100) |
|  | Oncomine comprehensive Assay plus (Thermo Fisher) +  Oncomine Focus Assay (Thermo Fisher) | 1 | 1 (100) |
| qPCR | AmoyDx MET Mutation Detection Kit (AmoyDx) | 1 | 1 (100) |
|  | Idylla GeneFusion Assay (Biocartis) | 4 | 4 (100) |
|  | EasyPGX ready ALK/ROS1/RET/MET  (Diatech Pharmacogenetics) | 2 | 2 (100) |
| NGS + qPCR | AmoyDx HANDLE Classic NGS Panel (AmoyDx) +  AmoyDx MET Mutation Detection Kit (AmoyDx) | 1 | 1 (100) |
|  | Oncomine Comprehensive Assay v3 (Thermo Fisher) + qPCR (keine Angabe) | 1 | 1 (100) |
|  | QIAseq Targeted RNAscan custom Panel (Qiagen) + AmoyDx MET Mutation Detection Kit (AmoyDx) | 1 | 1 (100) |
|  | AmpliSeq for Illumina Focus Panel (Illumina, DNA) +  Idylla GeneFusion Assay (Biocartis) | 1 | 1 (100) |

qPCR: Real-time PCR. NGS: Next generation sequencing. %: Success rate for each method used. No: Number
