# Supplementary material for: Pitfalls in Detecting MET Exon 14 Skipping Variants by DNA- and RNA-Based Next-Generation Sequencing Technologies in a Large Real-World Cohort and Results of the First Multinational External Quality Assessment Schemes
Source: J Mol Diagn. 2026 Jun 4;28(8):625–41. doi: 10.1016/j.jmoldx.2026.05.007 (PMC13494073; doi:10.1016/j.jmoldx.2026.05.007)
Supplement: Supplementary file 4 [file mmc4.docx]

**Supplemental Table S4: DNA-based methods used and results of the EQA sub-scheme with liquid biopsy samples in 2022.**

| **Manufacturer** | **Assay used** | **No. of participants** | **Participations with success (%)** |
| --- | --- | --- | --- |
| Agilent | Agilent SureSelect XT HS2 Custom Panel | 1 | 1 (100) |
| ArcherDX/IDT | Archer LIQUIDPlex ctDNA 28 Kit | 1 | 1 (100) |
|  | Archer Variantplex nNGM v2.0 | 1 | 0 (0) |
| Qiagen | QIAseq Targeted DNA Custom Panel | 2 | 1 (50) |
| Thermo Fisher  Scientific | Oncomine Lung cfDNA Assay | 1 | 0 (0) |
|  | Oncomine Precision Assay GX | 1 | 0 (0) |
|  | Oncomine Focus Assay | 1 | 0 (0) |

No: Number
