# Supplementary material for: Pitfalls in Detecting MET Exon 14 Skipping Variants by DNA- and RNA-Based Next-Generation Sequencing Technologies in a Large Real-World Cohort and Results of the First Multinational External Quality Assessment Schemes
Source: J Mol Diagn. 2026 Jun 4;28(8):625–41. doi: 10.1016/j.jmoldx.2026.05.007 (PMC13494073; doi:10.1016/j.jmoldx.2026.05.007)
Supplement: Supplementary file 5 [file mmc5.docx]

**Supplemental Table S5: DNA-based methods used and results of the EQA sub-scheme with liquid biopsy samples in 2024.**

| **Manufacturer** | **Assay used** | **No. of participants** | **Success rate (%)** |
| --- | --- | --- | --- |
| Agilent | Sure Select XT HS2 Custom Panel Lung Liquid | 1 | 1 (100) |
| ArcherDx/IDT | Archer VariantPlex Solid Tumor Kit | 1 | 1 (100) |
| ArcherDx/IDT | Archer LIQUIDPlex Genopath Solid Tumor Panel | 1 | 1 (100) |
| ArcherDx/IDT | Archer LIQUIDPlex Universal Solid Tumor Panel | 1 | 1 (100) |
| Hedera Dx | Profiling 2 ctDNA test panel | 1 | 1 (100) |
| Qiagen | QIAseq Targeted DNA Custom Panel | 3 | 2 (67) |
| Qiagen | QIAseq Targeted DNA Human Actionable Solid Tumor Panel | 1 | 0 (0) |
| Thermo Fisher Scientific | Oncomine Dx Express Test | 1 | 1 (100) |
| Thermo Fisher Scientific | Oncomine Precision Assay GX | 4 | 0 (0) |
| - | Lab developed test | 2 | 2 (100) |

No: Number
